# EiDA: A lossless approach for dynamic functional connectivity; application to fMRI data of a model of ageing

**DOI:** 10.1162/imag_a_00113

**Published:** 2024-03-22

**Authors:** Giuseppe de Alteriis, Eilidh MacNicol, Fran Hancock, Alessandro Ciaramella, Diana Cash, Paul Expert, Federico E. Turkheimer

**Affiliations:** Department of Neuroimaging, Institute of Psychiatry, Psychology and Neuroscience, King’s College London, London, United Kingdom; London Interdisciplinary Doctoral Programme, UCL Division of Biosciences, University College London, London, United Kingdom; Scuola Superiore Sant’Anna, Pisa, Italy; Global Business School for Health, University College London, London, United Kingdom

**Keywords:** ageing, fMRI, dynamic functional connectivity, brain network dynamics, LEiDA, phase-locking

## Abstract

Dynamic Functional Connectivity (dFC) is the study of the dynamic patterns of interaction that characterise brain function. Numerous numerical methods are available to compute and analyse dFC from high-dimensional data. In fMRI, a number of them rely on the computation of the instantaneous Phase Alignment (iPA) matrix (also known as instantaneous Phase Locking). Their limitations are the high computational cost and the concomitant need to introduce approximations with ensuing information loss. Here, we introduce the analytical decomposition of the iPA. This has two advantages. Firstly, we achieve an up to 1000-fold reduction in computing time without information loss. Secondly, we can formally introduce two alternative approaches to the analysis of the resulting time-varying instantaneous connectivity patterns, Discrete and Continuous EiDA (Eigenvector Dynamic Analysis), and a related set of metrics to quantify the total amount of instantaneous connectivity, drawn from dynamical systems and information theory. We applied EiDA to a dataset from 48 rats that underwent functional magnetic resonance imaging (fMRI) at four stages during a longitudinal study of ageing. Using EiDA, we found that the metrics we introduce provided robust markers of ageing with decreases in total connectivity and metastability, and an increase in informational complexity over the life span. This suggests that ageing reduces the available functional repertoire that is postulated to support cognitive functions and overt behaviours, slows down the exploration of this reduced repertoire, and decreases the coherence of its structure. In summary, EiDA is a method to extract lossless connectivity information that requires significantly less computational time, and provides robust and analytically principled metrics for brain dynamics. These metrics are interpretable and promising for studies on neurodevelopmental and neurodegenerative disorders.

## 1 Introduction

The brain is recognised as a complex dynamic system (Friston, 1997; Hancock, Rosas, Mediano, et al., 2022; Ladyman et al., 2013; Turkheimer et al., 2022) whose activity is best characterised by patterns of interaction across its constituent parts, at a multitude of scales, for example, molecular, cellular, systemic (D’Angelo & Jirsa, 2022). Consequently, dynamic functional connectivity (dFC) has become of major interest to understand brain function in health and disease (Cohen, 2018; Hutchison et al., 2013). dFC is an extension of functional connectivity (FC) (Fingelkurts et al., 2005), which aims to quantify the connectivity between signals in a functional sense, that is, where the connections are not anatomically informed, but based on a measure of similarity of the signal across the entire acquisition period (Friston, 2011; Stephan & Friston, 2009). Where FC is “static,” in the sense that it computes connectivity measures representative of the entire recording (e.g., Pearson Correlation), dFC analyses the time evolution of connectivity patterns (Arbabyazd et al., 2020; Calhoun et al., 2014; Cohen, 2018; Hansen et al., 2015; Hutchison et al., 2013).

In pursuing this objective, any dFC approach faces at least two challenges. The first pertains to the determination of the appropriate size of the observation window that has to be sequentially applied to the time-series (Hindriks et al., 2016). The second problem revolves around dimensionality as we are confronted with the task of analysing the temporal evolution of an N×N matrix (where N is the number of signals), which can present difficulties in terms of both analysis and lossless storage.

A commonly used solution for addressing the challenge of dimensionality is the so-called Leading Eigenvector Dynamic Analysis (LEiDA) (Cabral et al., 2017). In LEiDA, functional connectivity or phase coupling is calculated using the Hilbert transform. The Hilbert transform is a signal processing technique to convert a real signal into a complex valued analytical signal $A\left(t\right)e^{i\theta \left(t\right)}$, with an instantaneous amplitude A(t) and an instantaneous phase θ(t). The phase of the analytical signal is then extracted (Glerean et al., 2012) to generate the instantaneous Phase Alignment Matrix (iPA ) (also known as instantaneous Phase Locking) by computing the pairwise phase differences. This matrix is then decomposed into its orthogonal components, the eigenvectors, where the first or leading eigenvector is selected and the remaining discarded, thus reducing the dimensionality of the matrix from N×N to N × 1 dimensions. Clustering is then performed on the time-series of the first eigenvector to identify distinct and reproducible spatio-temporal patterns, or “modes” (sometimes referred to as “brain states”) of phase-alignment that the brain consistently exhibits throughout the recording period. The use of LEiDA has made important contributions to various functional brain paradigms, yielding insights across diverse areas of research, for example the study of sleep-wake transitions (Deco et al., 2019), the action of psychedelic drugs (Lord et al., 2019; Olsen et al., 2022), neurodevelopment (França et al., 2022), schizophrenia (Hancock, Rosas, et al., 2022), and depression (Figueroa et al., 2019; Martínez et al., 2020).

Using LEiDA, a number of dynamic indices of connectivity have been proposed such as the average duration of a mode, or its fractional occurrence. In addition, within modes, it is possible to define measures capturing ideas drawn from complex systems theory such as metastability, a metric reflecting simultaneous tendencies for global integration and functional segregation (Cavanna et al., 2018; Deco et al., 2017; Friston, 1997; Hancock, Cabral, et al., 2022).

However, three aspects of the current methodology are worth investigating. Firstly, one needs to quantify the information loss resulting from the retention of the leading eigenvector only (Cabral et al., 2017; Olsen et al., 2022). Secondly, there is a need to base current methodologies onto a formal mathematical characterisation of the iPA matrix—also to improve speed end efficiency of the algorithms (Hutchison et al., 2013). Thirdly, depending on the data, the methodology should be able to model the iPA matrix both as a granular set of dynamic modes as well as a smooth transition across FC configurations (Battaglia et al., 2020; Lavanga et al., 2023; Petkoski et al., 2023).

To address these challenges, we introduce EiDA (Eigenvector Dynamic Analysis). Given the limited rank structure of the iPA matrix (see Section 2), it is always possible to decompose it analytically into two eigenvectors, with complete null contribution of the remaining ones. This allows the compression of the N×N iPA matrix to N × 2 dimension, without any loss of information. Furthermore, using the analytical form of the two eigenvectors, it drastically reduces their computation time (up to 1000 times). Here, we demonstrate how the evolution of the two eigenvectors can be used to quantify the trajectories of the dynamic connectivity patterns. We used both a discrete state approach, using clustering of both the eigenvectors as in LEiDA, which we call Discrete EiDA, and a continuous flow analysis using a 2-dimensional embedding, which we call Continuous EiDA. Together with EiDA, we propose two theoretically informed measures of phase alignment based on the norm of the iPA matrix, namely the spectral radius and spectral metastability. All these measures can be applied to any dFC time-varying matrix, once the relevant number of eigenvectors is identified.

We applied EiDA to a longitudinal fMRI data-set acquired across the life-span of a cohort of rodents (four data-points; Brusini et al., 2022; MacNicol et al., 2022), to investigate the changes in brain network dynamics associated to ageing. This is a unique longitudinal study and an ideal set to test the robustness and reliability of EiDA as well as the utility of its derived metrics in understanding brain time-evolution or, as in this case, degeneration. Other studies have tackled this question (Battaglia et al., 2020; Chen et al., 2017), however without the availability of a longitudinal dataset, preventing the tracking of age-related changes in dFC.

## 2 Materials and Methods

**Fig. 1.**
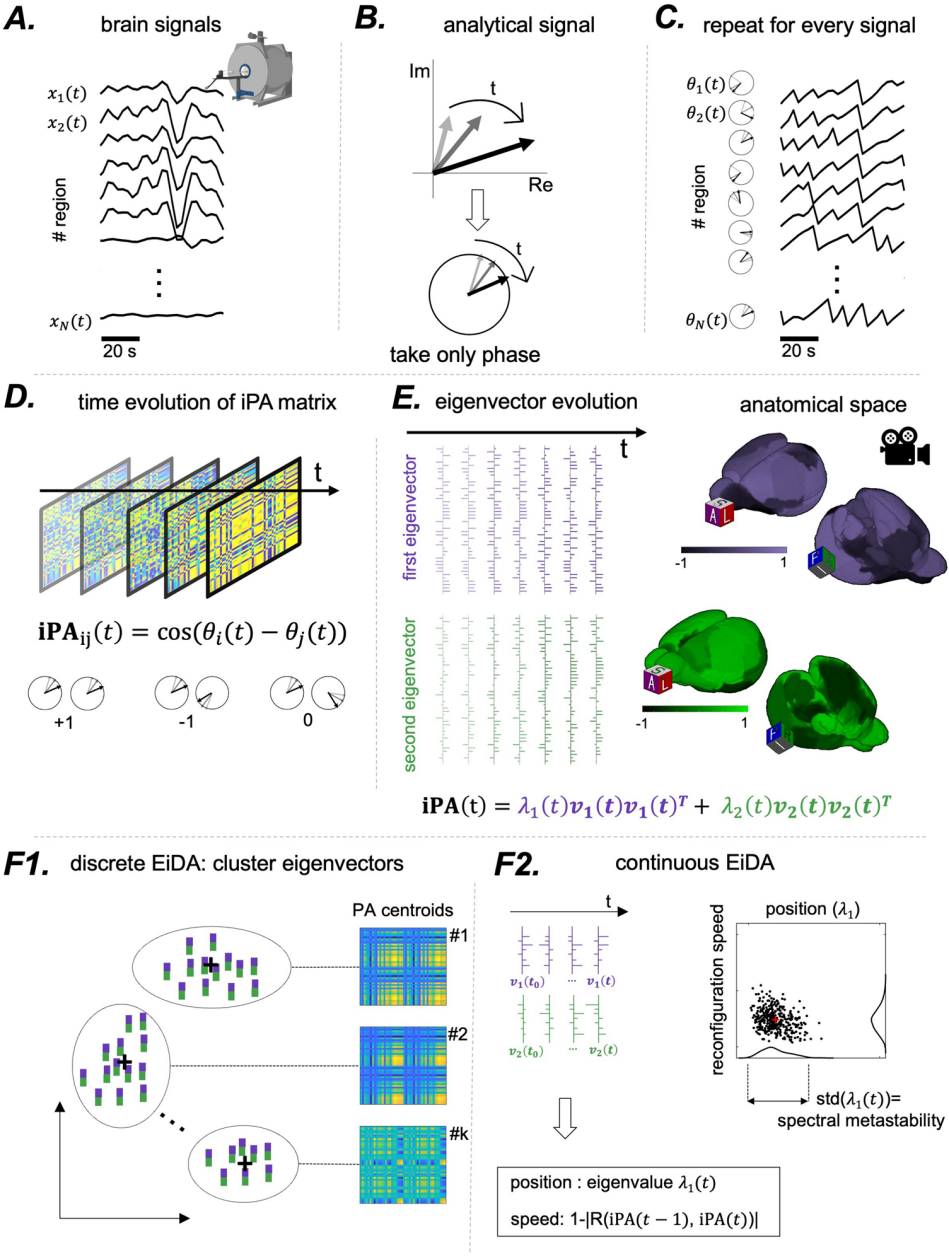
Summary of the proposed method. (A) We started from a set of N=44 -dimensional time-series, the rat fMRI signals from 44 brain parcels. (B) For each signal, we computed its analytical representation via the Hilbert Transform, obtaining a complex number evolving in time, of which we considered only the phase. (C) We repeated this procedure for every signal to obtain an N-dimensional time-series of phases. (D) At each time step, we computed the instantaneous Phase Alignment Matrix (**iPA**), obtaining a time-series of matrices. The entry i,j of the matrix is the cosine of the difference of the phases $\theta_{i}$ and $\theta_{j}$. If the two phases are equal, the cosine of the difference is 1, -1 if they are opposite, and 0 if they are in quadrature. (E) The matrix being of rank 2 (see Methods) it can be decomposed into two non-trivial eigenvectors, therefore reducing the data to a time-series of two vectors. Note that at each time t, the eigenvectors can be projected back in the anatomical space, as they have the same dimensionality as the orignal timeseries. (F) Once the time-series of eigenvectors for each recording are obtained, we propose two alternative analysis strategies. (F1) Discrete EiDA performs k-means clustering to identify k “modes.” See Section 2.8.1 for specifics about the algorithm. (F2) Continuous EiDA, on the other hand, embeds the flow of phase-alignment configurations in a 2D position-speed space; see Section 2.8.2. The first dimension is the first eigenvalue which is called “position,” because it is the norm of the **iPA** matrix. The second one is the speed at which the **iPA** matrix evolves, the reconfiguration speed. Alternatively, as in Figure 6, two position and speed plots can be computed using the two separate eigenvectors.

The main concepts of our analysis pipeline are outlined in Figure 1.

We defined a “recording” as the collection of N signals $x_{1}\left(t\right),x_{2}\left(t\right),...,x_{N}\left(t\right),t=1,\ldots ,T$ . We referred to a “group” as a set of recordings. In our case, as detailed in Section 2.11, the recordings are resting-state fMRI signals obtained during a 2-year study of brain ageing in rodents where the mean time-series was extracted from an anatomical parcellation of N=44 regions in each recording (Brusini et al., 2022; MacNicol et al., 2022). The recordings were repeated for each animal four times during the life-span of the study. The study is described in Section 2.11.

### 2.1 Static functional connectivity

We used static connectivity measures, that is, computed over the entire recording duration (Friston, 1994; Friston et al., 1993), as a point of reference for the dynamic investigations of the recordings. The simplest measure we considered was the Matrix of Pearson Correlations (PC) of the paired time-series. For each recording, we defined a single matrix P, where ${\text{P}}_{ij}=\text{R}(\;x_{i},x_{j})$ , and R(.,.) is the Pearson correlation coefficient between the signals i and j. To show the effect of ageing in the static correlation patterns, an overall inter-subject connectivity matrix for each of the four groups of recordings was obtained by averaging the squared coefficient values in each group, thus obtaining four mean FC matrices. In the averaging process, we calculated the squared values to take both positive and negative correlations into consideration. Additionally, we defined the FC Index as the sum of the squared values of the matrix (Demmel, 1997), as a synthetic measure of overall connectivity for a single recording.

### 2.2 iPA matrix

To perform a dynamic analysis and avoid the need to define time windows, one needs an instantaneous measure that can be used to compute the level of functional connectivity between each pair of brain regions. A common approach is to produce the analytical representation of a signal, which expresses a time series as a complex number, and thus an instantaneous amplitude A(t) and instantaneous phase θ(t). To compute the analytical signal, we used the Hilbert transform. The analytical form of a signal x(t) is equal to x(t)+iℋ{x(t)}, where ℋ{x(t)} is the Hilbert transform of the signal. The Hilbert transform of a signal is defined as:

$$ℋ\left\{x\left(t\right)\right\}=\frac{1}{\pi }\text{p.v.}\int_{-\infty }^{\infty }\frac{x\left(t\right)}{t-\tau }d\tau$$ (1)

For more details about the Hilbert transform, see Honari et al. (2021) and Bedrosian (1963). To provide a visual illustration (see Fig. 1), we can conceive the analytical signal as a “clock” with the hand of the clock that changes length over time (with the amplitude A(t)) and rotates (changes with phase θ(t)). Therefore, at each time instant t, one could ask whether two signals are “phase aligned,” that is, they have the same instantaneous phase θ(t): in this case, the two hands of the two clocks point in the same direction (see Fig. 1C,D). This can be done for each pair of signals, and at each time point. We can thus define an “instantaneous” phase alignment value $\text{iPA}{\left(\text{t}\right)}_{1,\;2}$ between two signals $x_{1}\left(t\right)$ and $x_{2}\left(t\right)$ as the cosine of the difference of two phases $\text{iPA}{\left(t\right)}_{1,2}=\text{cos(}\theta_{1}(t)-\theta_{2}(t))$ (Cabral et al., 2017; Deco et al., 2017; Figueroa et al., 2019; Hancock, Cabral, et al., 2022; Honari et al., 2021; Martínez et al., 2020; Vohryzek et al., 2020). This value is equal to 1 if signals are perfectly in phase, -1 if their phase difference is π, and 0 if their phase difference is $\pm \frac{\pi }{2}$, that is, the signals are in quadrature.

It is therefore possible to define, given N signals $x_{1}\left(t\right),x_{2}\left(t\right),...x_{N}\left(t\right)$, an instantaneous Phase Alignment Matrix, iPA Matrix, that is, a matrix where $\text{iP}{\text{A}}_{ij}(t)=$ $\text{cos(}\theta_{i}(t)-\theta_{j}(t))$ (Deco et al., 2017):

$$\text{iPA}\left(t\right)=\left[\begin{array}{cccc} \text{cos}\left(\theta_{1}\left(t\right)-\theta_{1}\left(t\right)\right) & \text{cos}\left(\theta_{1}\left(t\right)-\theta_{2}\left(t\right)\right) & \ldots & \text{cos}\left(\theta_{1}\left(t\right)-\theta_{N}\left(t\right)\right)\\ \vdots & \ddots & & \vdots \\ \text{cos}\left(\theta_{N}\left(t\right)-\theta_{1}\left(t\right)\right) & \text{cos}\left(\theta_{N}\left(t\right)-\theta_{2}\left(t\right)\right) & \ldots & \text{cos}\left(\theta_{N}\left(t\right)-\theta_{N}\left(t\right)\right) \end{array}\right]\in {\mathbb{R}}^{N\times N}$$ (2)

The analysis of connectivity patterns over time is transposed to the analysis of the evolution of the iPA matrix over time. Note, however, that modeling signals as oscillators with an instantaneous phase is meaningful only if signal are narrow-band. This is the case in our data (see Section 2.11).

To compare static and dynamic connectivity matrix, we computed the average iPA matrix for each recording, and then calculated the FC Index as in Section 2.1.

### 2.3 Analytical computation of the eigenvectors of iPA matrix

Let us consider the N signals at a certain time t and their instantaneous phases, computed via Hilbert Transform, which form a vector $\vec{\theta }\left(t\right)={\left(\theta_{1}\left(t\right),\;\theta_{2}\left(t\right),\;\theta_{3}\left(t\right),\;\;.\;\;.\;\;.\;\;\theta_{N}\left(t\right)\right)}^{T}$ $\in {\mathbb{R}}^{N}$. From this vector, we define, as in the previous section, an instantaneous Phase Alignment (iPA ) matrix, which is in principle different at each time t. Let us now, for simplicity, consider a single time t, and call the matrix iPA by abuse of notation, but remembering that the $\vec{\;\theta }$ vector changes at every time t and so does the matrix. Hence, the next procedure can be repeated at each time t.

Given that the matrix $\text{iP}{\text{A}}_{ij}=\text{cos(}\theta_{i}-\theta_{j})$ , and given that cos(x−y)=cos(x)cos(y)+sin(x)sin(y) , then $\text{iP}{\text{A}}_{ij}=\text{cos(}\theta_{i})$ $\text{cos(}\theta_{j})+\text{sin(}\theta_{i}\text{)sin(}\theta_{j})$ . This means that the iPA matrix can be decomposed in the sum of two matrices:

iPA=[cos(θ1)cos(θ1)…cos(θ1)cos(θN)⋮⋱⋮cos(θN)cos(θ1)…cos(θN)cos(θN)]+[sin(θ1)sin(θ1)…sin(θ1)sin(θN)⋮⋱⋮sin(θN)sin(θ1)…sin(θN)sin(θN)] (3)

We define the two vectors, the “cosine” vector $\text{c}={\left(\text{cos(}\theta_{1}),\text{cos(}\theta_{2}),...,\text{cos(}\theta_{N})\right)}^{T}\;\in {\mathbb{R}}^{N}$, and the “sine” vector $\text{s}={\left(\text{sin(}\theta_{1}),\text{sin(}\theta_{2}),...,\text{sin(}\theta_{N})\right)}^{T}\in {\mathbb{R}}^{N}$, and rewrite the matrix as $\text{iPA}=\text{c}{\text{c}}^{T}\;+\text{s}{\text{s}}^{T}$.

This matrix is symmetric and has ones on the diagonal, as $\text{iP}{\text{A}}_{ii}=\text{cos}\left(\theta_{i}-\theta_{i}\right)=\text{cos}\left(0\right)=1$ . Its trace is thus Tr(iPA)=N. Importantly, this decomposition demonstrates that it is a matrix with at most rank 2, which, being symmetric and positive semidefinite, will have no more than 2 non-null, positive eigenvalues $\lambda_{1}$ and $\lambda_{2}$ and their associated eigenvectors $v_{1}$ and $v_{2}$ (as observed by Olsen et al., 2022). This also implies that $Tr\left(\text{iPA}\right)=N=\lambda_{1}+\lambda_{2}$, as the sum of the eigenvalues of a matrix is equal to the trace of the matrix. Moreover, the two non-trivial eigenvectors will be a linear combination of c and s (as also observed by Olsen et al., 2022), so they will be of the form v=Ac+Bs . Thus, the computation of the two scalar values, A and B, is all that is required to solve the eigenvalue equation iPA(Ac+Bs)=λ(Ac+Bs) and determine the corresponding eigenvalues and eigenvectors:

$$\begin{array}{cc} \begin{array}{c} \text{iPA}\left(A\text{c}+B\text{s}\right)=\left(\text{c}{\text{c}}^{T}+\text{s}{\text{s}}^{T}\right)\left(A\text{c}+B\text{s}\right)=\\ \;\;\;\;\;\;\text{c}{\text{c}}^{T}A\text{c}+\text{c}{\text{c}}^{T}B\text{s}+\text{s}{\text{s}}^{T}A\text{c}+\text{s}{\text{s}}^{T}B\text{s}=\\ \;\;\;\;\;{\left\|\text{c}\right\|}^{2}A\text{c}+{\text{c}}^{T}\text{s}B\text{c}+{\text{s}}^{T}\text{c}A\text{s}+{\left\|\text{s}\right\|}^{2}B\text{s}=\\ \;\;\;\;\;\;\;\;\;\;\;\;\;\;\;\;\;\;\;\;\;\;\;\;\;\;\;\;\;\gamma A\text{c}+\xi B\text{c}+\xi A\text{s}+\sigma B\text{s} \end{array} & \end{array}$$ (4)

Having renamed the following quantities:

$$\begin{array}{cc} \begin{array}{c} \gamma ={\left\|\text{c}\right\|}^{2}\\ \sigma ={\left\|\text{s}\right\|}^{2}\\ \xi ={\text{c}}^{T}\text{s}={\text{s}}^{T}\text{c} \end{array} & \end{array}$$ (5)

We can then highlight similar terms in the eigenvalue equation as (γA+ξB)c+(ξA+σB)s=λAc+λBs . In the general case of linear independence of components, we have a system of two equations and two unknowns:

$$\{\begin{array}{c} \gamma \;A+\xi \;B=\lambda \;A\\ \xi \;A+\sigma \;B=\lambda \;B \end{array}$$ (6)

This represents an equivalent and reduced eigenvalue problem in two dimensions:

$$\left(\begin{array}{cc} \gamma & \xi \\ \xi & \sigma \end{array}\right)\left(\begin{array}{c} A\\ B \end{array}\right)=\lambda \left(\begin{array}{c} A\\ B \end{array}\right)$$ (7)

With associated eigenequation $\lambda^{2}-\left(\sigma +\gamma \right)\lambda +\sigma \gamma -$ $\xi^{2}=0$ . Defining $\Delta ={\left(\sigma -\gamma \right)}^{2}+4\xi^{2}$ and observing that σ+γ=N, we can express the solutions as:

$$\begin{array}{cc} \begin{array}{c} \lambda_{1}=\frac{N+\sqrt{\Delta }}{2}\\ \lambda_{2}=\frac{N-\sqrt{\Delta }}{2} \end{array} & \end{array}$$ (8)

Finally, respective normalised eigenvectors are:

$$\begin{array}{cc} \begin{array}{c} \left(\begin{array}{c} A_{1}\\ B_{1} \end{array}\right)=\left(\begin{array}{c} 1\\ \frac{\left(\sigma -\gamma \right)+\sqrt{\Delta }}{2\xi } \end{array}\right)\Rightarrow {\text{v}}_{1}=\frac{\text{c}+B_{1}\text{s}}{\left\|\text{c}+B_{1}\text{s}\right\|}\\ \left(\begin{array}{c} A_{2}\\ B_{2} \end{array}\right)=\left(\begin{array}{c} 1\\ \frac{\left(\sigma -\gamma \right)-\sqrt{\Delta }}{2\xi } \end{array}\right)\Rightarrow {\text{v}}_{2}=\frac{\text{c}+B_{2}\text{s}}{\left\|\text{c}+B_{2}\text{s}\right\|} \end{array} & \end{array}$$ (9)

In conclusion: $\text{iPA}=\lambda_{1}{\text{v}}_{1}{\text{v}}_{1}T+\lambda_{2}{\text{v}}_{2}{\text{v}}_{2}T$.

### 2.4 Two cases of interest

This analytical decomposition allows us to understand the iPA matrix structure and the role of its two eigenvectors and respective eigenvalues. To do so, let us consider two cases.

**Fig. 2.**
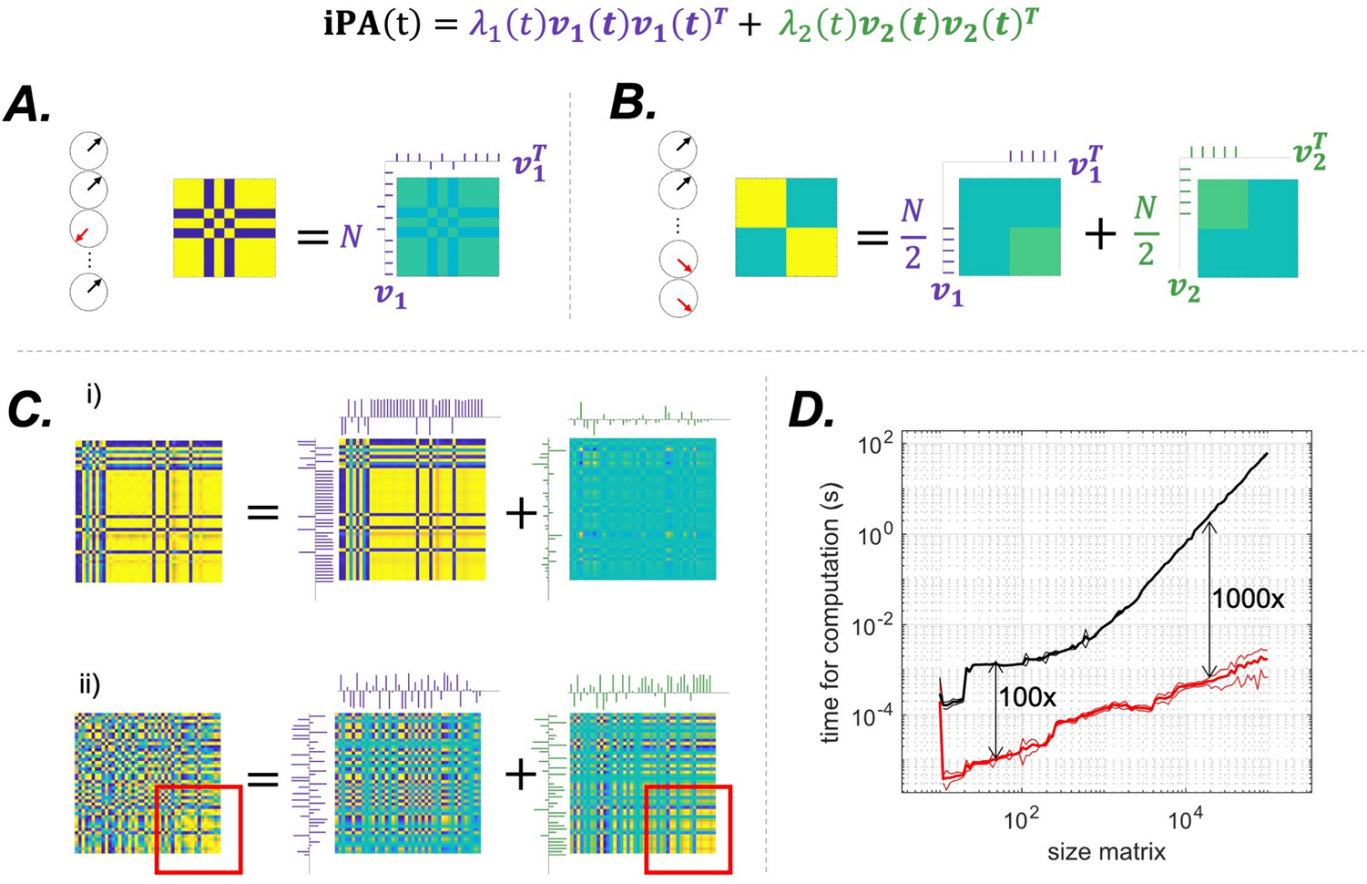
Illustration of the eigenvalue decomposition of limit cases matrices in (A) and (B) corresponding to $\lambda_{1}=N$ and $\lambda_{1}=\lambda_{2}$, and instructive exemplars from the rat data in (C). Purple and green ticks indicate the first and second eigenvectors respectively. (A). Rank 1 matrix: all signals are either in phase or in anti-phase, as indicated by the clocks. The matrix can be decomposed into a single eigenvector, with maximum eigenvalue $\lambda_{1}=N$ . (B) Both eigenvectors are equally important: the first N/2 signals are in quadrature with the second N/2. The two blocks of the matrix are reconstructed by summing 50% of the first eigenvector and 50% of the second. The eigenvalues are both equal to N/2. In this case, discarding one eigenvector would result in losing half of the information contained in the iPA matrix. (C) Two interesting cases from our data. In the first one (i), the value of the first eigenvector is high, $\lambda_{1}=39$ out of a maximum of 44, and contains most of the information. It is noticeable that in this case almost all signals are either in phase or in anti-phase, as can be seen from the large blocks. In the second case (ii), $\lambda_{1}=23$ , close to N/2 = 22. In this case, the two eigenvectors contain almost the same amount of information. Throwing away the second one would lead to a large error in the reconstruction of the matrix: the information contained in area highlighted by the red square is contained in the second eigenvector. (D) Average speed in seconds (mean, continuous line, plus or minus one standard deviation, dotted line) of our algorithm (red line) and the gold standard algorithm for iPA analysis (LEiDA), as a function of the size of the iPA matrix in a log log plot. Black arrows indicate that the black line is 100 or 1000 times the red one.

The first case is $\lambda_{1}=N$ , which implies $\lambda_{2}=0$ . The iPA matrix then has rank 1, as it only has 1 non-null eigenvalue; see Figure 2A. In this case, the c vector is parallel to the s vector. A rank 1 matrix with ones on the diagonal must have all elements equal to plus or minus one, that is, this is the trivial case where all signals are in phase or antiphase: $\vec{\theta }=(\theta_{0}+\left(2k_{1}+1\right)\pi ,\theta_{0}+\left(2k_{2}+1\right)\pi ,\theta_{0}+\left(2k_{3}+1\right)$ ${\pi ,...\theta_{0}+\left(2k_{N}+1\right)\pi )}^{T},k_{1...N}\in \mathbb{Z}$ . Then, the matrix is maximally rank deficient and contains minimal information. Physiologically, this is the case of maximally aligned/anti-aligned phases, which manifests with the first eigenvalue $\lambda_{1}$ tending to N.

The second case is when $\lambda_{1}=\lambda_{2}=N/2$, see Figure 2B. Given the constraints on the iPA matrix, this is possible if and only if c and s are orthogonal, their norms are both equal to N/2, which is proven as follows. Given that the eigenvalues differ by $\sqrt{\Delta }$, we must impose Δ=0 and therefore ξ=0 and σ=γ . This means that c and s are orthogonal and they have same norm, which is $\gamma =\sigma =N/2=\lambda_{1}=\lambda_{2}$. Note that if they are orthogonal it means that the two eigenvectors are c and s themselves, because $\text{iPAc}=(\text{c}{\text{c}}^{T}+\text{s}{\text{s}}^{T})\text{c}=\text{c}{\text{c}}^{T}\text{c}=\gamma \;\text{c}$ and similarly iPAs=σs .

Therefore, when $\lambda_{1}=\lambda_{2}$ the information contained in the iPA matrix is maximally irreducible to a single eigenvector, and therefore there is no “leading” eigenvector as the connectivity pattern is fully expressed by two orthogonal components which are both equally important, as the relative magnitude of the eigenvalues represents their contribution to the total information contained in the iPA matrix. An example of this configuration is a four-block matrix where the two diagonal blocks are ones, all signals in phase, and the two non-diagonal blocks are zero, all signals in quadrature; see Figure 2B.

These analytical considerations on the structure of the iPA matrix allow us to define the following measures:

### 2.5 First eigenvalue = spectral radius

The iPA matrix is uniquely characterised by its first eigenvalue $\lambda_{1}$, because $\lambda_{2}=N-\lambda_{1}$ as it is a rank 2 matrix. $\lambda_{1}$ is called the spectral radius of the iPA matrix, which is a norm of the matrix. Hence, it is a closed form norm of the instantaneous Phase Alignment matrix. Indeed, as it tends to N, this norm indicates that all signals tend to the “trivial” state where they all are in phase or antiphase (see Section 2.4). At the same time, the information contained in the matrix and its complexity are reduced in this case, because iPA becomes maximally rank deficient and so maximally ordered. On the other hand, the more the norm approaches N/2 , the less the matrix is irreducible to a single connectivity pattern and therefore one sees a less ordered phase alignment pattern.

In conclusion, $\lambda_{1}$ is a measure of both the amount of connectivity and the informational complexity of iPA .

### 2.6 Spectral metastability

The spectral radius can be considered as a global information metric of the instantaneous phase alignment of the signals, expressed in closed form, fast to calculate, and conceptually similar to the Kuramoto Order Parameter (Cabral et al. 2011; Deco et al., 2017; Hancock, Cabral, et al., 2022; Hancock, Rosas, McCutcheon, et al., 2022; Hancock, Rosas, Mediano, et al., 2022; Lord et al., 2019; Shanahan, 2010; Wildie & Shanahan, 2012). Note that the Kuramoto order parameter is the average phase of a system, while $\lambda_{1}$ takes the iPA matrix, so all the pairwise interactions, into account. Therefore, we can define a new general measure of metastability, the standard deviation over time of $\lambda_{1}$, which is also equal to the standard deviation of $\lambda_{2}$ as $\lambda_{2}=N-\lambda_{1}$. We call this measure “spectral metastability” as the first eigenvalue is the spectral radius of the iPA matrix.

$$\text{met}{\text{a}}_{spec}=\text{std(}\lambda_{1})=\text{std(}\lambda_{2})$$ (10)

Therefore, spectral metastability is a measure of variability in the exploration of connectivity patterns and reflects simultaneous tendencies for coupling and decoupling.

### 2.7 Irreducibility index

The total information in the matrix is the sum of two orthogonal components, the eigenvectors, scaled by the two eigenvalues. If the first eigenvalue is lower than a given percentage of $N=\lambda_{1}+\lambda_{2}$, this implies that the reduction of the iPA matrix to the first eigenvector would keep less than this percentage of the total information. Firstly, we need to define a threshold representing the minimum amount of information one wants to be explained if we used only the first eigenvector, expressed as a percentage of N. The Irreducibility Index is then the proportion of time during the recording in which the first eigenvalue of the iPA matrix is lower than the predefined threshold. If the experimenter requires to keep at least x% of the information at each time step, the Irreducibility Index at a level x% indicates the fraction of the recording in which using only the first eigenvector would fail in keeping this information. The higher the Irreducibility Index reflects the more information is lost, at a given threshold, by using the only first eigenvector.

### 2.8 Two new approaches to analyse eigenvector dynamics

The sections above demonstrated that the N×N iPA matrix can be fully and without loss of information decomposed into two eigenvectors of size N and one eigenvalue. These form the basis for two approaches to analyse the dynamics of eigenvectors and eigenvalues over time. The first one is called “Discrete EiDA,” because it finds k discrete states by clustering the eigenvectors in line with the approach of the original LEiDA. The second is called “Continous EiDA,” because it interprets the reconfiguration of eigenvectors as a continuous trajectory and quantifies its overall “position” and “speed” in a 2D space. Based on dynamical considerations on the evolution of eigenvectors, as we will do in Section 3.1, the experimenter can choose to use the first or the second approach.

#### 2.8.1 Discrete EiDA

Discrete EiDA is a new k-means-based algorithm that clusters the information contained in the two eigenvectors. Our proposed approach is mathematically equivalent to performing k-means with the full **iPA** matrices. However, it exploits the fact that the information of the matrix is fully contained in its two eigenvectors. Therefore, it operates on the eigenvectors instead of the full matrices, allowing to compute k-means in an efficient way, and to store only the two eigenvectors instead of the full matrices. This is relevant as demonstrated in Section 3: in the data considered here, discarding the second eigenvector would neglect a significant amount of information, as indicated by the Irreducibility Index (defined in Section 2.4).

Each data-point entered into the algorithm is an iPA matrix, represented in a condensed form by its two non-trivial eigenvectors. Discrete EiDA iterates between two steps: the first step assigns each data-point to its closest k centroids. The second step updates the centroids by computing the mean of each cluster of data points.

The k centroids are saved as the centroid iPA matrices in the upper triangular form, which is computationally feasible since k is a small number, while the data points are stored in a dataset D as stacked eigenvectors weighted with the square roots of eigenvalues. In the first step, all the distances are computed by rebuilding the matrix from the eigenvectors ($\text{iPA}=\lambda_{1}{\text{v}}_{1}{\text{v}}_{1}T+\lambda_{2}{\text{v}}_{2}{\text{v}}_{2}T$) and computing the cosine distance between the upper triangular part of the rebuilt matrix and the centroid matrix. Then, in the second step, the centroid matrices are updated by averaging the upper triangle of the rebuilt matrices. Overall, we are clustering the full information contained in the matrices, without any loss, but we are using only the two non-trivial eigenvectors.

This is the pseudo-code for Discrete Eida:

**Table:** 

**Algorithm 1:** Discrete EiDA K-means
1: **procedure** Discrete Eida (dataset D, number of clusters K) 2: Initialise K centroids randomly represented as upper triangular matrices 3: Assign each data-point to the centroid with smallest EidaDistance (data-point, centroid) 4: **repeat** 5: Update the centroids by ComputeMean (each cluster) 6: Assign each data-point to the centroid with smallest EidaDistance (data-point, centroid) 7: **until** No data point reassignments 8: **return** Cluster assignments and centroids 9: **function** ComputeMean (cluster) 10: Initialise *total* as an upper triangular matrix with all zeros 11: **for** i←1 to size of cluster **do** 12: Take data-point at index i in cluster 13: Rebuild $\text{iPA}=\lambda_{1}{\text{v}}_{1}{\text{v}}_{1}T\text{​}+\lambda_{2}{\text{v}}_{2}{\text{v}}_{2}T$ 14: Take the upper triangular part of the rebuilt matrix 15: sum the upper triangular rebuilt matrix to *total* 16: $\begin{array}{l} \text{return}mean\text{ as}\;total\text{ divided by the size of }\\ \text{the}\;\text{cluster} \end{array}$ 17: **function** EidaDistance(data-point, centroid) 18: rebuild iPA from the two eigenvectors by the formula $\text{iPA}=\lambda_{1}{\text{v}}_{1}{\text{v}}_{1}T+\lambda_{2}{\text{v}}_{2}{\text{v}}_{2}T$ 19: take the upper triangular part of the iPA 20: **return** the cosine distance between the centroid and the upper triangular part

The final output of Discrete EiDA is k clusters with k centroids, which are identified with k phase-alignment brain modes. The clustering of the first eigenvectors was already proposed in previous approaches (Cabral et al., 2017; Figueroa et al., 2019; Hancock, Cabral, et al., 2022; Lord et al., 2019; Martínez et al., 2020; Vohryzek et al., 2020). Here instead, we propose to consider both eigenvectors because, as we have seen, the rank 2 iPA makes it possible to cluster the full information contained in the matrix in a lossless and efficient manner. Once the clusters are computed, it is then possible to visualise their representative states, the centroids, as averaged iPA matrices in the clusters. We highlight that since the centroids are the average of multiple rank-2 iPA matrices, their rank will no longer be 2. Indeed, they contain the full information on the average connectivity pattern in the clusters. Moreover, this new algorithm solves a known problem in the LEiDA literature, the flipping sign problem (Hancock, Rosas, McCutcheon, et al., 2022). Because eigenvectors have a sign ambiguity (an eigenvector is still an eigenvector if its sign is inverted), clustering in the leading eigenvector space poses a problem as two opposing sign eigenvectors representing the same state would cancel each other. Various approaches have been proposed to solve the flipping-sign problem (Olsen et al., 2022); however, it is worth noticing that rebuilt matrices and their combinations are invariant to the flipping-sign of the eigenvector; therefore, with Discrete EiDA we solve the root of the problem by working in the native space of symmetric positive semidefinite iPA matrices, instead of the leading eigenvector space. Moreover, when $\lambda_{1}\approx \lambda_{2}$, the first and the second eigenvector may switch. Again, for the same reasons, Discrete EiDA is robust to this problem.

Moreover, once the clusters are computed, it is possible to use synthetic measures of the duration of modes (the same defined in LEiDA (Cabral et al., 2017; Figueroa et al., 2019; Lord et al., 2019; Martínez et al., 2020; Vohryzek et al., 2020), in particular:

1) The Fractional Occurrence of a brain mode, that is, the relative amount of time in the recording in which the iPA matrix belongs to a specific mode or cluster.
2) The Dwell Time, that is, the average duration of a mode or cluster.

These two measures are not equivalent: a mode could appear very frequently in a recording, that is, have a high fractional occurrence, but be on average for a very brief time, that is, have a low dwell time.

Finally, within each cluster, it is possible to compute any dynamic measure of interest like spectral metastability (see Section 2.8), similarly to what was done in Hancock, Rosas, McCutcheon, et al. (2022), or to apply all the continuous measures introduced in Section 2.8.2.

However, we underline that a wide range of clustering methods exist beyond k-means clustering and might lead to different clusters, and they might be preferable depending on data, their distributions, and the experimental question. Therefore, we consider EiDA as a method that uses the eigenvector representation of the phase alignment information to find a discrete set of brain modes, where the clustering method is essentially a free parameter.

It is important to underline that we do not expect the brain dynamics to present a clear and distinctive number of clusters (k). Hence, we believe that a better conceptualisation of the centroids is their use as masks to interpret brain dynamics and obtain time-varying iPA information metrics (like fractional occurrence, dwell time, or metastability) rather than as real neurobiological states, particularly as they can be dependent on the specific clustering method used. This is in line with the description offered by Probabilistic Metastable Substates, which describes the brain dynamical state as a set of recurrent patterns of phase coherence, or substates, which occur with given probabilities (Deco et al., 2019; Kringelbach et al., 2020).

It is well documented that the clusters found in data are dependent on the method employed; see, for example, the performance of different state-of-the-art methods on benchmarks (Shang et al., 2022). For example, k-means clustering would yield centroids which are averaged **iPA** matrices, while k-medoids clustering would yield centroids which are actual **iPA** matrices which are “central” in the multi-dimensional data cloud, which would likely be, even if marginally, different.

#### 2.8.2 Continuous EiDA

The clusters may not always be well separated due to a lack of space heterogenetiy of the cloud distribution of the eigenvectors. In this case, the continuous exploration of phase alignment configurations may be analysed by plotting the dynamic walk of connectivity motifs in a 2D embedding (Nicolau et al., 2011; Saggar et al., 2018).

Therefore, we introduce Continuous EiDA, an approach that examines the evolution of both eigenvectors over time as a continuous flow.

To do so, we propose to follow the time evolution of iPA in a two-dimensional “position-speed space,” using both eigenvectors in a kinematic speed-displacement (KSD) plot (Meriam, 1971).

The two dimensions in this 2D embedding are: the “position” p(t) (overall amount of connectivity at time t), and the “speed” of evolution of the iPA matrix s(t). The position is what the previous sections have demonstrated to be the best summary indicator of the state of the phase alignment matrix: the spectral radius = first eigenvalue $\lambda_{1}$. The speed is the “reconfiguration speed” (as already proposed by Battaglia et al., 2020; Hancock, Cabral, et al., 2022), at which the matrix evolves in its space, computed as the correlation distance between temporally adjacent matrices in their upper triangular form. We define reconfiguration speed s(t) as:

s(t)=1  −|R( iPA(t), iPA(t−1))| (11)

where R is the Pearson Correlation Coefficient.

This is a 2D embedding of the evolution of the iPA matrix. However, the individual evolution of each of the two eigenvectors may be also interesting to understand how the two orthogonal components of the iPA matrix change with time. It is possible to apply the same embedding for the two eigenvectors separately. In this case, the “position” will be the eigenvalue associated to the eigenvector, that is, $\lambda_{1}$ for $v_{1}$ and $\lambda_{2}$ for $v_{2}$, and the “speed” will be the same as Equation 11 but computing the correlations of the eigenvectors instead of the full iPA matrix.

#### 2.8.3 Functional connectivity dynamics

The EiDA framework also allows one to compute Functional Connectivity Dynamics (FCD). FCD is a tool to visualise the dynamic repertoire of **iPA** (Hansen et al., 2015). Taking the reconfiguration speed as the distance between a matrix and a matrix at the previous timestep, FCD is a matrix with all the possible pairwise distances: its entries ij are the distance between $\text{iPA}(t_{i})$ and $\text{iPA}(t_{j})$ . To compute it, we use the same distance proposed in the reconfiguration speed:

$$\text{FC}{\text{D}}_{ij}=1\;\;-|\text{R}(\text{iPA}(t_{i}),\text{iPA}(t_{j})\text{)|}$$ (12)

FCD allows therefore to observe the switching behaviour of the brain in a unique time-to-time distance matrix. We can compute then measures of dynamical fluidity, like the mean of FCD (Petkoski et al., 2023) or variance of FCD (also known as Switching Index) (Lavanga et al., 2023).

### 2.9 An alternative measure of phase synchronisation

A commonly used measure of metastability can be obtained from one of the most popular measures of synchronisation, the Kuramoto Order Parameter

$$r=\frac{1}{N}\sum_{i=1}^{N}e^{i\theta_{i}(t)}$$ (13)

that can be computed for each time $\theta_{1}\left(t\right)$, using the phases of the analytical signals (Cabral et al. 2011; Deco et al., 2017; Hancock, Cabral, et al., 2022; Hancock, Rosas, McCutcheon, et al., 2022; Hancock, Rosas, Mediano, et al., 2022; Lord et al., 2019; Shanahan, 2010; Wildie & Shanahan, 2012).

The modulus of r∈[0,1] is a measure of synchronisation. If all signals are in phase, it will be 1, while, if they are distributed uniformly, it will be 0. Metastability is defined as the standard deviation of the modulus of the Kuramoto order parameter over time (Cabral et al., 2011; Deco et al., 2017; Hancock, Cabral, et al., 2022; Hancock, Rosas, McCutcheon, et al., 2022; Hancock, Rosas, Mediano, et al., 2022; Lord et al., 2019; Shanahan, 2010; Wildie & Shanahan, 2012), and we refer to it as Kuramoto metastability to clearly differentiate it from spectral metastability introduced above.

$$\text{met}{\text{a}}_{kop}=\text{std(}\left|r(t)\right|)$$ (14)

### 2.10 Informational complexity

We calculated a proxy for the informational complexity of the evolution of the eigenvectors, computing the number of bits required for compression using the Lempel–Ziv–Welch (LZW) algorithm (Welch, 1984; Ziv & Lempel, 1978). A higher complexity, reflected by the need to use more bits to store information, indicates more random and unpredictable patterns, while a lower complexity indicates more coherent and structured patterns of evolution with less information to store. Similar approaches have been introduced to quantify perturbational complexity of EEG responses to Transcranial Magnetic Stimulation (TMS) (Casali et al., 2013).

### 2.11 Application of EiDA to rat fMRI data

We applied the methods described above to a longitudinal dataset of ageing rats (Brusini et al., 2022; MacNicol et al., 2022, MacNicol et al., in preparation). A cohort of 48 Sprague Dawley rats (Charles River, UK) were monitored across their lifespan and scanned up to four times with a 9.4 T Bruker Biospec MR scanner, specifically at 3, 5, 11, and 17 months of age. These ages represent, respectively, late adolescence, young adulthood, middle age, and the beginning of senescence (Sengupta, 2013). Experiments were performed in accordance with the Home Office (Animals Scientific Procedures Act, UK, 1986) and approved by the King’s College London’s ethical committee. Resting-state functional data were recorded using a 2D multi-slice multi-echo echo planar imaging sequence with TR = 2750 ms, TEs = 11, 19, 27, and 35 ms, and a $70^{{}^{\circ}}$ flip angle, producing an image with 40 slices. Slices were 0.5 mm thick with a 0.2 mm gap, which gave a 48×44 matrix, with an in-plane resolution of 0.5 x 0.5 mm. Rats were anaesthetised with ca. 1.8% isoflurane for the duration of functional scans. This dose produced anatomically-plausible components from single-subject and group-level Independent Component Analysis (ICA) (Beckmann & Smith, 2005; MacNicol, 2021).

The multi-echo acquisition was split into sub-volumes for each echo time. Motion correction to the volume temporally halfway through the acquisition (i.e., the middle volume) was estimated using only the sub-volume with the earliest echo time. Each echo volume was realigned identically by applying the generated parameters. The corrected echo volumes were optimally combined, which maximises the signal-to-noise ratio at the expense of some loss-of-time resolution (DuPre et al., 2021). Signals were simultaneously filtered with a 0.01–0.08 Hz band-pass filter and nuisance factors that included motion parameters and the CSF signal. Corrected and filtered fMRI volumes were warped to a study-specific template (MacNicol et al., 2022) and parcellated into 44 anatomical regions of interest (ROI), 22 for each hemisphere, generated by combining delineations of predominantly grey matter structures from two popular rat atlases (Papp et al., 2014; Valdés-Hernández et al., 2011). The BOLD signals were averaged within each ROI.

The application of Hilbert transform and the construction of the iPA matrix relies on the assumption that signals are narrow-band, which is the case given the bandpass filter we applied (Cabral et al., 2017; Hancock, Cabral, et al., 2022). A qualitative comparison of group-level ICA showed no substantial change in the spatial distribution of the components compared to the 0.01–0.1 Hz bandpass filter, which is commonly applied to data from rodents under isoflurane anaesthesia (Grandjean et al., 2023; Hamilton et al., 2017; Jonckers et al., 2014).

### 2.12 Statistical analysis

We restricted our analyses to the 30 rats that were scanned four times. As not all the measures were normally distributed (Anderson-Darling normality tests, see Supplementary Material), we used non-parametric one-way ANOVA test (Kruskal-Wallis test) to analyse the four time-points. A post-hoc Wilcoxon rank sum test was then used to test the variability between each pair of age groups. The multiple comparison correction was performed by controlling the False Discovery Rate (FDR) at a rate α=0.05 , using the Benjamini-Hochberg procedure (Benjamini & Hochberg, 1995). The detailed p-values and ANOVA tables for all the measures are reported in the Supplementary Material. In the Figures, we report significance only for the consecutive pairs of age groups, given that the measures are usually increasing/decreasing curves.

A Pearson Correlation Coefficient R was used to test collinearity between measures where the test for significance was obtained by calculating an empirical null R distribution by shuffling data.

## 3 Results

### 3.1 Static functional connectivity

**Fig. 3.**
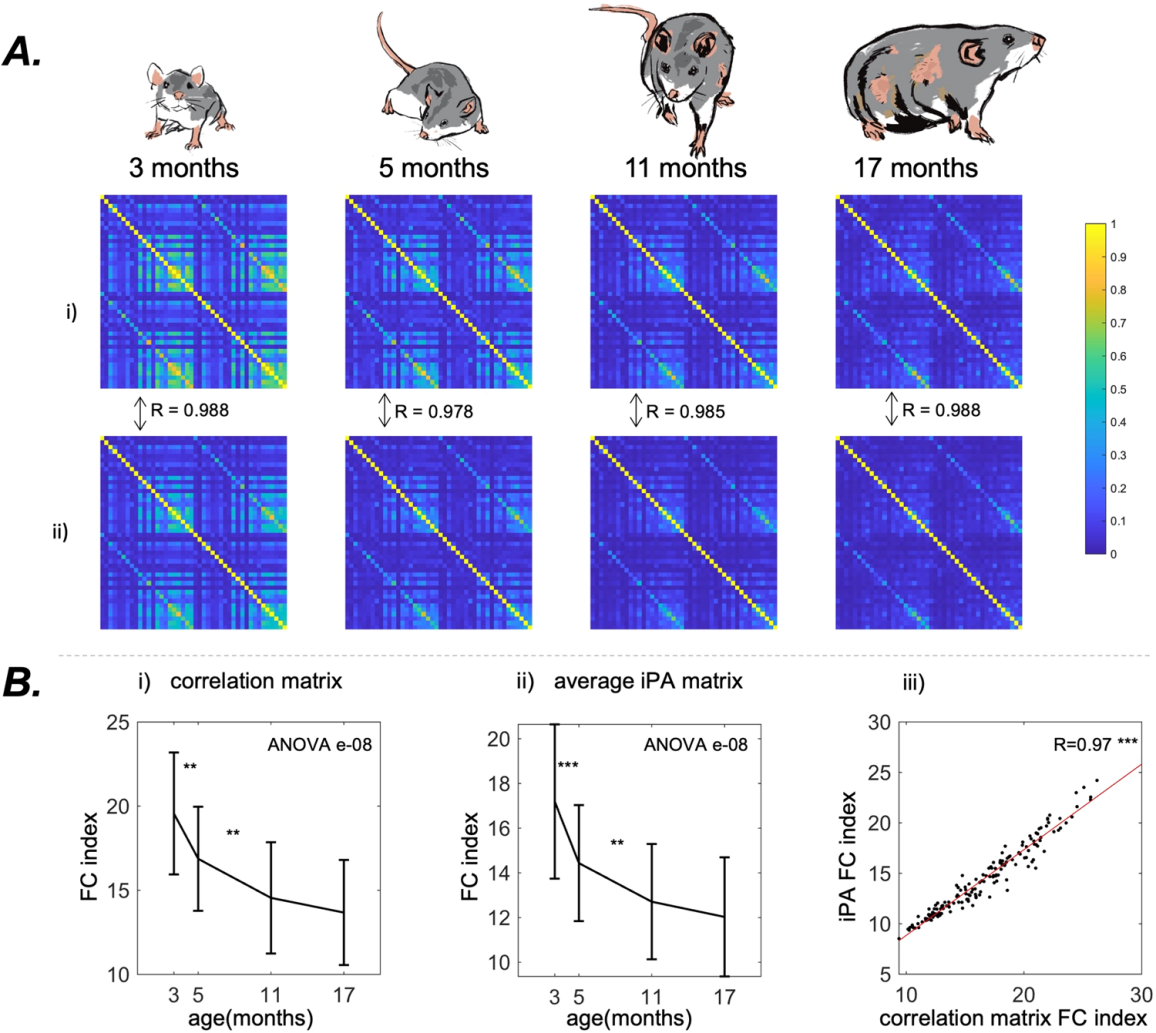
Results from the static analysis (A). The group-averaged Pearson connectivity matrix (i) and time-averaged iPA matrix (ii) in the four age groups. We indicate the element-wise correlations between the Pearson correlation matrices and the iPA matrices. Ageing is associated with an overall decrease of connected hubs (the bright yellow hubs of correlations). (B) The evolution with ageing (mean +- standard deviation) of the FC Index (see Section 2.1) for Pearson correlation matrices (i) and for the averaged iPA matrices (ii). Both the measures confirm a decrease which demonstrates the loss of connectivity strength observable in (A). (iii): Correlation between the two measures with the best Ordinary Least Squares fit. The two measures are highly correlated, suggesting that the average iPA matrix conveys the same information as the Pearson correlation matrix. P-value of the nonparametric ANOVA test is reported inside the figures. * indicates p < 0.05, ** p < 0.01, and *** p < 0.001 in the paired Wilcoxon tests between adjacent time points. Asterisks are reported only if p-values pass the Benjamini-Hochberg correction.

A loss of total correlation and a diminution of the number of correlated areas over time is visible in the mean FC matrices for both PC and iPA connectivity (3.1 A). The FC Index (see Section 2.1) sharply decreases from 19.7 ± 3.9 at month 3 to 13.7 ± 3.1 at month 17 with an overall significance of e-08 (nonparametric ANOVA) in PC connectivity, with similar statistically significant decreases (ANOVA p = e-08) for iPA . The correlation between the FC Indexes of PC and iPA connectivity was both statistically significant (p < 0.001) and high (R = 0.97) (Fig. 3).

### 3.2 Dynamic functional connectivity

#### 3.2.1 Computation times

We measured the time required to compute the eigenvalue decomposition of the iPA matrix and compared our approach with the benchmark algorithm for LEiDA, implemented in Matlab, by varying the matrix size logarithmically from N=10 to $N=10^{5}$ and generating 20 iPA matrices for each size. As shown in Figure 2D, with our computer (Intel(R) Xeon(R) Silver 4116 CPU @ 2.10 GHz), EiDA is 100 times faster for matrices with dimensions of 100 x 100, and becomes 1000 times faster for matrices larger than 10,000 x 10,000. Note that the matrix decomposition in the two eigenvectors also allows to save each matrix with 2N elements instead of $\frac{N\left(N-1\right)}{2}$ , offering a significant advantage in terms of RAM requirements. Computational speed should ease the extension of EiDA to higher dimensionality in both space and time.

#### 3.2.2 Information loss

We computed the information loss when considering just the first eigenvector at different thresholds. Figure 5C shows the Irreducibility Index with four thresholds of 70, 65, 60, and 55%. The Irreducibility Index with a threshold of 65% is greater than 0.4 in 3 months rats and becomes greater than 0.7 in 17 months rats. This means that discarding the second eigenvector would neglect a significant (more than 35%) amount of functional connectivity information over the life-span of the rats. The same considerations hold for the Irreducibility Index with thresholds of 60 and 70%. Information loss always increases with age.

### 3.3 Dynamic functional connectivity measures: discrete EiDA

We performed Discrete EiDA clustering for each of the age groups separately with k (number of clusters) from 1 to 10. We plotted the sum of squared distances of each data point in a cluster with their centroids as a function of k to obtain an “elbow plot.” We therefore obtained four elbow plots, one for each age group (see Supplementary Fig. S8). The point where this curve presents an elbow is often chosen as the optimal number of clusters because it indicates that adding another cluster would not significantly improve the clustering performance. We found k = 3 as a satisfactory partitioning of the space for all the age groups. As specified in Section 2.8.1, we interpret the centroids of the spatiotemporal patterns as masks to analyse the dynamic reconfiguration of phase alignment patterns and to compute dynamic measures like dwell time, fractional occurrence, or metastability (Hancock, Cabral, et al., 2022). As such, we apply clustering for interpretation and quantification of brain network dynamics.

**Fig. 4.**
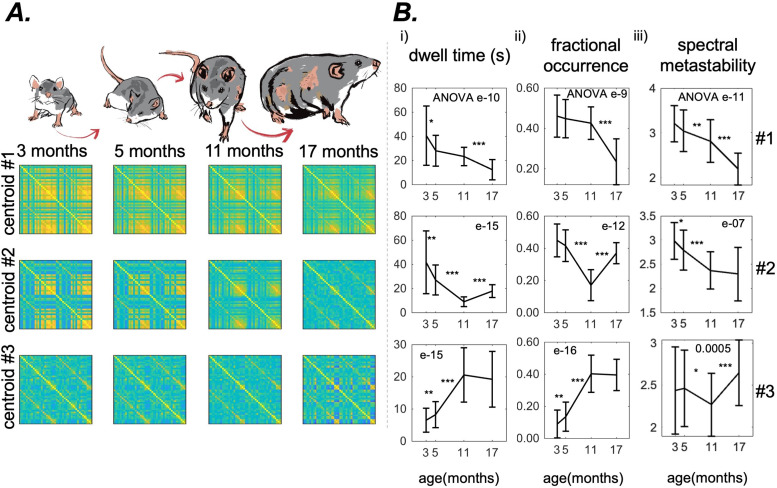
Results for the Discrete EiDA (A) The three EiDA cluster centroids and their evolution with ageing. Each row represents a centroid, each column a different age group (B) the evolution with ageing of (i) dwell time and (ii) fractional occurrence of clusters. The first two clusters, that are associated with connected hubs and reflect the connectivity information contained in the Pearson correlation matrices, become less frequent in the recordings as rats age. On the other hand, the third cluster, which is associated with a less structured and weaker connectivity pattern, increases in both dwell time and fractional occurrence with ageing. (iii) Spectral metastability in the three clusters. This measure decreases with ageing in the first two clusters, while there is not a clear pattern in the third. P-value of the nonparametric ANOVA test is reported inside the figures. * indicates p < 0.05, ** p < 0.01, and *** p < 0.001 in the paired Wilcoxon tests between adjacent time points. Asterisks are reported only if p-values pass the Benjamini-Hochberg correction.

The three centroids for the four age groups are shown in Figure 4A. For an expanded representation of the centroids as matrices with labels for brain regions see Supplementary Figures S1-S3. For a representation of the centroids as chord plots see Supplementary Figures S4-S7. We observe a gradual reduction in functional connectivity across all centroids as a function of age.

Dwell time of the first cluster shortened from 43.4 ± 28 s to 12.4 ± 8 s (ANOVA p = e-10); fractional occurrence from 0.46 ± 0.1 to 0.23 ± 0.1, (ANOVA p = e-9). In the third cluster, dwell time increased from 6.4 ± 4 s to 19.3 ± 9 s, (ANOVA p = e-15); fractional occurrence from 0.1 ± 0.1 to 0.4 ± 0.1 (ANOVA p = e-16).

On the other hand, the second cluster presented a decrease in dwell time (from 43.4 ± 27 s to 9.7 ± 4 s) and fractional occurrence (from 0.45 ± 0.1 to 0.18 ± 0.1) from to 3 to 11 months, and an increase of these measures (dwell time to 17.9 ± 5 s and fractional occurrence to 0.37 ± 0.1) from 11 to 17 months.

Spectral metastability of the first two clusters decreased with ageing, going from 3.2 ± 0.4 to 2.2 ± 0.4 (ANOVA p = e-11) for the first and from 3.0 ± 0.4 to 2.3 ± 0.6 for the second (ANOVA p = e-7). We did not observe a clear pattern of increase/decrease in spectral metastability of the third cluster.

### 3.4 Dynamic functional connectivity measures: continuous EiDA

**Fig. 5.**
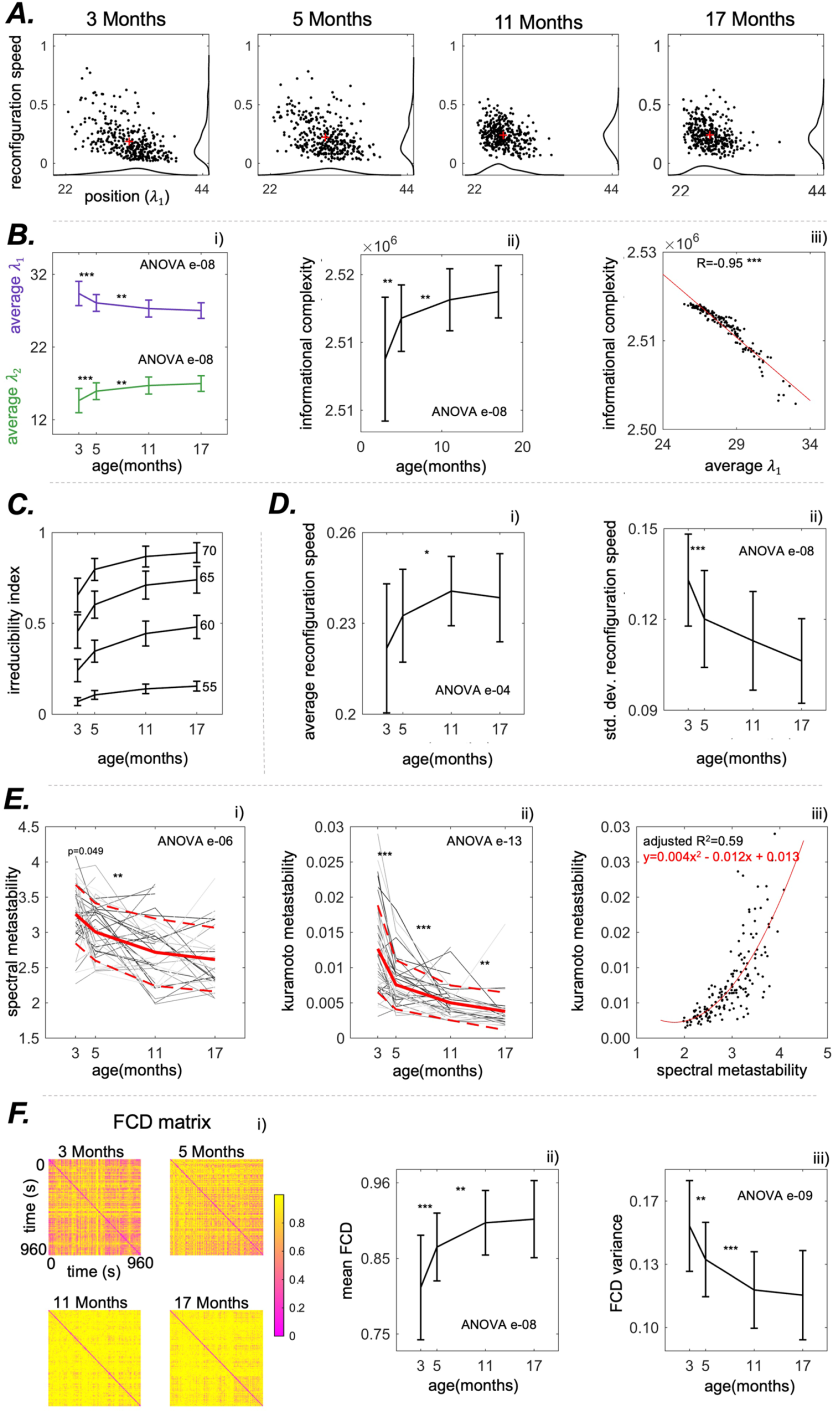
Results for the Continuous EiDA (A). The position-speed evolution in a representative rat. The position is the first eigenvalue of the iPA matrix, and the speed is the overall reconfiguration speed of the matrix (considering both eigenvectors). Red crosses represent the centers of mass of the distributions. Patterns evolve from a broader exploration of the space to a more compact one with age, where the first eigenvalue decreases and reconfiguration speeds grows. This means that connectivity patterns over time start from a more structured regime (less speed, higher eigenvalues) to a more random one. (B) The measures of interest: (i) average eigenvalue per recording (mean ± variance, first eigenvalue in purple, second in green). The first eigenvalue shows a decrease over time, in line with the loss of structure that is noticeable in 6B. (ii) informational complexity (iii) correlation between informational complexity and first eigenvalue, reflecting that the higher the first eigenvalue, the lower the amount of information contained in the **iPA** matrix. (C) Irreducibility Index, we plot four thresholds: 70, 65, 60, and 55%. Plot has no significance indicated because we are not interested in the effect of ageing but in the fact that a non-negligible amount of the recordings is not irreducible to a single eigenvector. Moreover, for visual clarity, mean ± half of the variance is shown. (D) (i) Average reconfiguration speed of the iPA matrix: it evolves faster with ageing (ii) standard deviation of the reconfiguration speed, quantifying the more compact exploration of the space associated with ageing. (E). The evolution with ageing of (i) metastability and (ii) spectral metastability for all the N=48 rats. Red lines indicate means, and dotted lines indicate standard deviations. Here, the individual trajectories (gray lines) of each single rat are shown. Some lines present discontinuities in case the recording for a specific rat and age group was missing. Ageing is associated with a significant decrease of both Kuramoto metastability and spectral metastability (iii) Scatter plot of spectral metastability versus Kuramoto metastability. The red line represents the quadratic fit that was performed to explain metastability as a function of spectral metastability. (F) (i) the FCD matrices in the same representative rat. The FCD is a time-to-time matrix (see Methods): it contains the distances of the iPA matrices for each pair of time points. (ii) Mean of the FCD matrix as a function of age. (iii) Variance of the FCD matrix as a function of age. P-value of the nonparametric ANOVA test is reported inside the figures. * indicates p < 0.05, ** p < 0.01, and *** p < 0.001 in the paired Wilcoxon tests between adjacent time points. Asterisks are reported only if p-values pass the Benjamini-Hochberg correction.

The position and speed plots for the iPA matrix in a representative rat are shown in Figure 5A. Trajectories demonstrated significant changes with ageing. Patterns started as trajectories with a broad exploration of the configuration space and finished confined to a more compact area. Moreover, as measured below, the trajectories of younger rats exhibited a higher contribution from the first eigenvector.

#### 3.4.1 Order and information complexity

As shown in Figure 5B.i, the average of the first eigenvalues per recording shows a statistically significant decay with ageing (from 29.5 ± 1.8 to 27.0 ± 1.0, ANOVA p = e-08). As in the case of static connectivity, this decay plateaued from 11 months to 17 months, suggesting that there is no significant change from middle age to senescence. We observed an increase (ANOVA p = e-08) in the Informational Complexity measure defined in 2.9 with ageing that indicates that connectivity configurations evolve in a less structured way in older rats compared with younger rats, as in Figure 5B.ii. We correlated informational complexity with the average value of the first eigenvalue per recording, obtaining an overall correlation of -0.95, p < 0.001 (Fig. 5B.iii). This establishes an information theoretical relation between the first eigenvalue and the amount of information contained in the iPA matrix, as also explained in Section 2.4.

#### 3.4.2 Reconfiguration speed

We noticed an age-related increase in the average reconfiguration speed of iPA (Fig. 5D.i.) from 0.22 ± 0.02 to 0.24 ± 0.01 (ANOVA p = e-04) and a decrease in the standard deviation of the reconfiguration speed (Fig. 5D.ii.), from 0.13 ± 0.02 to 0.11 ± 0.01 (ANOVA p = e-08).

#### 3.4.3 Exploration of the spatiotemporal configuration space

From the kinematic speed-displacement plots in Figure 5A, we observed that as the rats age, there is a reduced exploration of the position-speed space. The decrease is quantified by the standard deviation of the reconfiguration speed and spectral metastability (standard deviation of “position”). We found that spectral metastability declines with age from 3.2 ± 0.4 to 2.6 ± 0.5 (ANOVA p = e-06) (Fig. 5.E.i). Kuramoto metastability shows the same pattern of decay related to age. Indeed, spectral metastability and Kuramoto metastability are related. Through a quadratic fit, we found that the relation between spectral metastability (x) and the Kuramoto metastability (y) is the following: $y=0.004x^{2}-0.012x+0.013$ , adjusted R squared = 0.59 (Fig. 5.E.iii). In conclusion, older brains are characterised by less variability both in the order of the iPA matrix and in its rate of change.

#### 3.4.4 Informational differences between the two eigenvectors

We then applied Continuous EiDA to the two eigenvectors separately, as explained in Section 2.8.2, gaining insight into the dynamic structural changes of the iPA matrix and its two building blocks. This offers a way to analyse brain network dynamics in a dimensionally reduced space, without any loss of information.

**Fig. 6.**
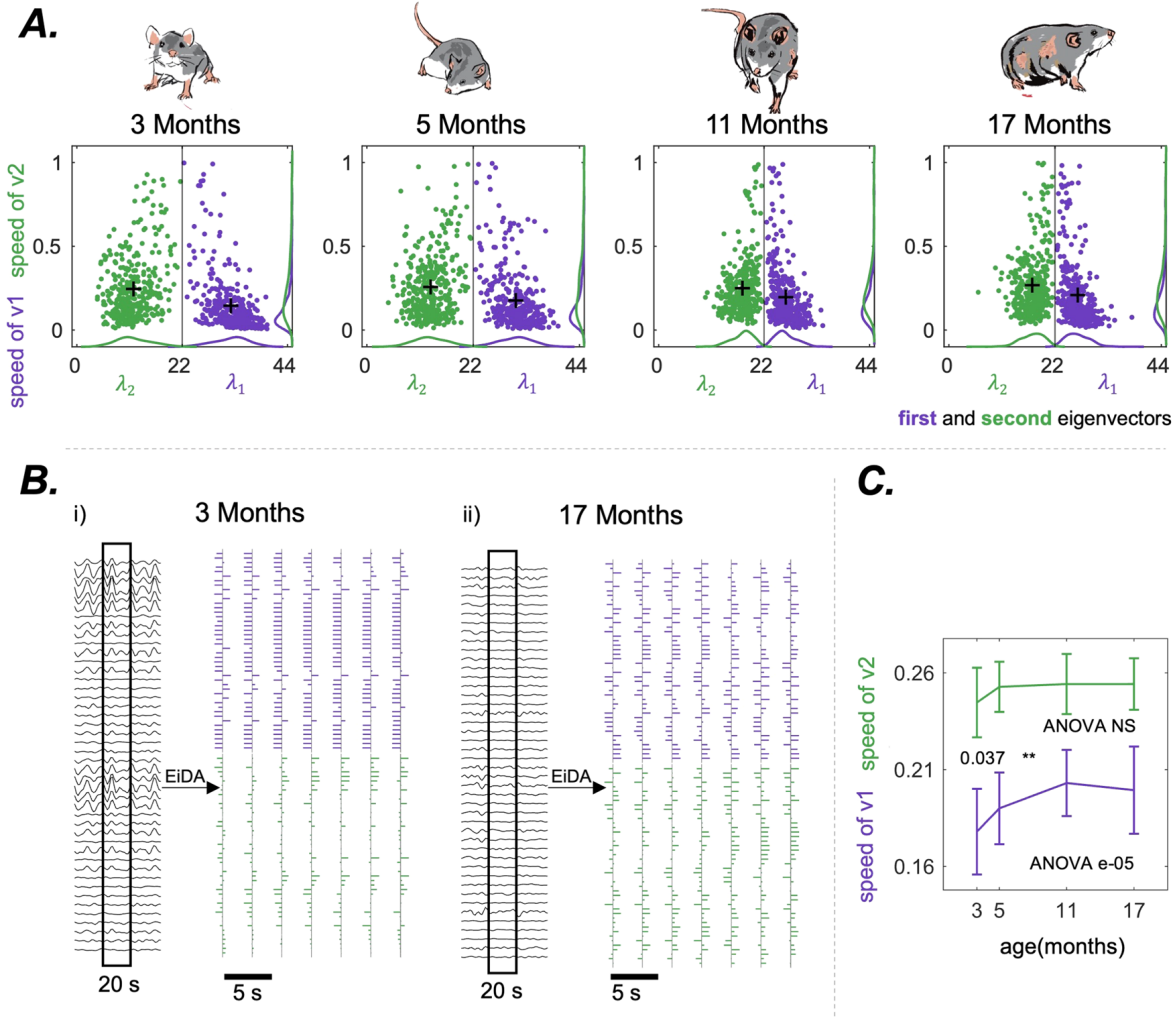
(A) The position-speed plots of the two separate eigenvectors, for the same representative rat as in Figure 5A. Here, the position for each eigenvectors is its associated eigenvalue, and the speed is the reconfiguration speed of the eigenvector. (B) The EiDA transformation from the raw data to the evolution of the two eigenvectors for the same representative rat of (A) in two different cases: 3 months (left) and 17 months (right). Note that in the first case we can observe higher instantaneous phase alignment in the raw signals. This is related to a more ordered and compact evolution of the eigenvectors, and the fact that the first eigenvector contains most of the information. In the second case, there is a lack of instantaneous phase alignment. This is reflected by more random, less ordered, and equally informative eigenvectors when considering their respective loadings. (C) Average reconfiguration speed of first (purple) and second (green) eigenvector. We observe an increase in the speed of the first eigenvector. P-value of the nonparametric ANOVA test is reported inside the figures. * indicates p < 0.05, ** p < 0.01, and *** p < 0.001 in the paired Wilcoxon tests between adjacent time points. Asterisks are reported only if p-values pass the Benjamini-Hochberg correction.

Figure 6A shows in the same representative rat of Figure 5A, the 2D embedding for the evolution of the two eigenvectors. Figure 6B shows the transformation from the raw data to the eigenvector evolution using EiDA, in a specific portion of the recording, for 3 months and 17 months. At 3 months, signals are highly phase aligned, as observable in the data. This is indicated by a more compact and ordered evolution of both eigenvectors: there is a clear dominant pattern of phase alignment (first eigenvector, almost all brain areas in phase). On the other hand, in the 17 months case, both eigenvectors evolve more randomly and without a dominant underlying pattern.

We observed (Fig. 6C) a significant (ANOVA p = e-05) increase in the reconfiguration speed of the first eigenvector with ageing, from 0.18 ± 0.02 to 0.20 ± 0.02. Hence, ageing is associated with a speed-up in the “walk” of the dominant pattern of connectivity. Notably, the evolution of the second eigenvector is always faster than the evolution of the first, as also visible in Figure 6B. We also found that the reconfiguration speed of the overall matrix is strongly positively correlated with the reconfiguration speed of the first eigenvector (R = 0.902, p < 0.001), and positively correlated with the reconfiguration speed of the second eigenvector (R = 0.674, p < 0.001). Finally, we found a decrease in the FCD variance (ANOVA e-09) and an increase of the mean FCD (ANOVA e-09); see Figure 5.

These results show that EiDA provides a formal mathematical characterisation of the **iPA** matrix that allowed the extraction of complementary metrics through consideration of brain dynamics as both a granular set of dynamic modes as well as smooth transitions across FC configurations.

## 4 Discussion

In this study, we investigated state dynamics and reconfiguration dynamics on resting-state fMRI data from a study of ageing in rats using EiDA that provides an analytically based framework for dimensionality reduction and dynamical analysis. EiDA’s high speed and memory efficiency may enable advancements that would have been computationally unfeasible so far, for example, real-time algorithms (Gui et al., 2022; Lorenz et al., 2021; Váša et al., 2022) or studies with a high number of brain signals, where parcellation is extremely fine-grained or absent. Here, we showcase its utility from a number of theoretical and application perspectives.

### 4.1 Eigenvector decomposition of the iPA matrix

EiDA provides an analytical solution expressible in closed form for the lossless decomposition of the instantaneous Phase Alignment matrix. Complete information is encoded in two eigenvectors and one eigenvalue, allowing the calculation of conventional dynamic metrics and the derivation of novel measures to further characterise the dynamic evolution of the spontaneous activity in the resting-state brain. Additionally, the analytical methods significantly accelerate the calculation of the orthogonal decomposition and enable its extension to much higher dimensional data, in time and/or space.

### 4.2 Role of the eigenvalues and eigenvectors of the iPA matrix

As EiDA is an eigenvector decomposition of the iPA matrix, one could be tempted to interpret the eigenspace spanned by first eigenvector as the denoised form of the matrix. However, this may not be necessarily true as there is no clear differentiation of signal and noise, particularly in the regime where $\lambda_{1}$ is far from its maximum possible value N. One could articulate an interpretation of the leading eigenvector as the container of the large-scale information about the connectivity patterns, while the second eigenvector contains more localised details, as in Figure 6B.i and Figure 2C.i., following ideas from graph signal analysis (Expert et al., 2017). This interpretation is possible only if the first eigenvalue is significantly higher than the second. This interpretation breaks down in the regime where $\lambda_{1}\approx \lambda_{2}$ as shown by the counter-example of Figure 2B, where in a fully structured matrix, made of four blocks, the two eigenvalues are equal, and using only the first eigenvector would discard 50% of the overall phase alignment information. In conclusion, both eigenvectors and eigenvalues are necessary to interpret the iPA matrix.

The analytical derivation in this paper allows the rigorous definition of a global parameter summarising the heterogeneity in the signal phases, the first eigenvalue or spectral radius of the iPA matrix, whose distribution over time contains the information both on global connectivity and on informational complexity of the matrix; the standard deviation of this distribution we have defined as a novel estimator of metastability.

This eigenvalue can be a more accurate indicator than the Kuramoto Order Parameter of the phase alignment structure, being the norm of the phase alignment matrix, rather than the center of mass of the instantaneous angles. For example, in a maximally synchronised and ordered state, as in Figure 2A, signals in antiphases would cancel out in computing the Kuramoto Order Parameter, while the first eigenvector would reach its maximal possible value and capture the “singular” structure of this configuration. Moreover, the first eigenvalue can be computed in any possible matrix (e.g., the sliding window correlation matrix), making the EiDA measures more general.

### 4.3 Dynamical and complexity insights for ageing

The results of static analysis show a decrease of connectivity through ageing (FC Index). The dynamic analysis corroborates and expands on this. Application of the novel approach revealed a decrease in the average spectral radius, spectral metastabilty, and variability of reconfiguration speed together with an increase of informational complexity, as rat brain activity evolved over their life-span. Additionally, the time spent in each state together with its fractional occurrence changed as the rats aged.

Discrete EiDA captured the changes in spatiotemporal patterns of connectivity associated with age. These patterns showed a loss of phase synchrony and a reduced connectivity structure with ageing. This likely explains the increase in fractional occurrence and dwell times of the third centroid, that is associated with disrupted brain synchrony, and the decrease of fractional occurrence and dwell times of the first two centroids, which relate to phase-aligned states. This is also in line with previous results from ageing studies obtained using different methodologies, exploring both static and dynamic functional connectivity (Andrews-Hanna et al., 2007; Battaglia et al., 2020; Betzel et al., 2014; Escrichs et al., 2021; Salat, 2011; Zimmermann et al., 2016). This increase in randomness is also shown by the loss of structure of the second centroid at 17 months (Fig. 4A). This means that at the age of 17 months there is a rearrangement of centroids when connectivity breaks down with ageing. This explains also why fractional occurrence and dwell time of the second cluster increase again at the age of 17 months, after having decreased. Changes in the third centroid may also be associated with a notable decline in the segregation of brain systems observed with ageing. Specifically, this decline manifests as an enhanced global communication pattern at the expense of localised “within-network” interactions, which, in turn, is linked to a reduction in specialised brain activity. This phenomenon has been attributed to the overall deterioration in the quality and processing of sensory input and the generation of motor output, leading to an increase in more abstract and loosely associated cognitive operations (Chan et al., 2014; Kawagoe, 2022).

Our interpretable 2D embedding, with “position” representing overall connectivity and “speed,” allowed us to visualise the effect of ageing on the exploration of dFC patterns. Older brains are less flexible, reflected by less variability in the exploration of connectivity patterns and in the speed of the exploration: the “filling” of the space becomes more compact with age. Both Continuous EiDA applied to the full iPA matrix and to the separate eigenvectors, with their associated measures, highlighted this result. Indeed, a decrease in spectral metastability, both in the overall recordings and in the separate EiDA clusters, was observed. Metastability is related to cognitive flexibility and adaptability to the environment (Hellyer et al., 2015), and the decrease we found is in line with previous findings, where a proxy measure of metastability was computed based on the temporal variability of intrinsic ignition or integration (Escrichs et al., 2021). This decrease is also confirmed by the decrease in the FCD variance, which is another measure of variability in the exploration of connectivity patterns. This is interestingly in line with recent human findings (Lavanga et al., 2023). Chen and co-authors (Chen et al., 2017) also demonstrated a reduced variability of dynamic functional connectivity in fMRI data with age.

The increase of the reconfiguration speed of the first eigenvector and the increase of the mean FCD associated with ageing is also intriguing as the brain dynamics is shown to move from a relatively coherent exploration of the kinematic space to a more random exploration with ageing, as pointed out by Nobukawa et al. (2019) and Petkoski et al. (2023). The increase in reconfiguration speed when considering the full **iPA** matrix appears to be in conflict with findings in human ageing studies (Battaglia et al., 2020; Petkoski et al., 2023). There are several possible explanations for these discrepancies. First, we compute the *instantaneous* reconfiguration speed and not windowed speeds. Second, our study subjects are rats and not humans, and in contrast to human studies, the rats are anaesthetised. When we compare the average reconfiguration speeds using the full **iPA** matrix (Fig. 5D) with the speeds from the individual eigenvectors (Fig. 6C), we find that the statistically significant increase is only found in the first eigenvector. In other words, the global dynamics slow down while the local interactions remain constant. This appears to corroborate previous studies which found that healthy ageing in humans was associated with a switch in the local versus global interaction balance (McIntosh, 2019; McIntosh et al., 2014).

It is also worth noticing that the general worsening of dynamic and information metrics happens while rat brains increase in mass with ageing (MacNicol et al., 2022); this is the opposite of what happens in humans as brains slowly decrease their mass after maturation. However, in rats, the metabolic rate of glucose per unit mass decreases with age (Le Poncin-Lafitte & Rapin, 1980) and increase in mass corresponds to a progressive reduction in the total number of neurons that starts also around the third month (Morterá & Herculano-Houzel, 2012). We postulate that the decrease in metastability could be related to these metabolic changes, and we plan to address this question in future studies.

Continuous EiDA showed also an increase of the dynamic complexity of dFC. Increases in complexity with healthy ageing have previously been demonstrated using point-wise correlation dimension (Müller & Lindenberger, 2012) and multi-scale entropy (McIntosh, 2019; McIntosh et al., 2014) in resting-state neuroimaging which is congruent with our findings. Taken together, our findings are particularly compelling as similar results are obtained using two distinct methods grounded in different theoretical frameworks: dynamical system analysis and information theory. Furthermore, by using our analytical interpretation and by correlating eigenvalues with informational complexity, we have established a link between dynamic connectivity analysis and the notion of informational complexity of signals.

Retaining only the leading eigenvector (as in LEiDA) appears to result in age-related increase in dFC information loss. This implies that, in ageing, more information is encoded in the second eigenvector. This is interesting as one can thus interpret the second eigenvector to be more related to the localised connectivity information (Expert et al., 2017). Accordingly, previous studies (McIntosh, 2019; McIntosh et al., 2014) have shown that healthy ageing is associated with a shift in the local versus global balance, with less information coded in global interactions and more in the local dynamics.

A potential caveat in our interpretation may be the choice of temporal filters. However, this is an intrinsic problem in all dFC approaches. The Hilbert transform requires selecting a (narrow) bandpass filter, with the risk of losing meaningful information. However, other approaches, like sliding-window correlation matrices, rely on the definition of a window of observation of the signals that limits the range of observable frequencies. Differences between rodent fMRI studies can be exacerbated if significant contributions to the BOLD signal are excluded by a filter that is too narrow (Grandjean et al., 2014). This study, like other small animal MRI studies, relied on an anaesthetic protocol that shifts the BOLD signal’s frequency distribution (Paasonen et al., 2018). However, in our case, we did not observe any substantial differences in a qualitative comparison of the spatial distribution of group ICA components. Nonetheless, this work primarily provides a proof-of-concept, so future work may identify an optimal narrow-pass window and the degree to which, if at all, temporal filtering impacts the stability of these results.

## 5 Conclusion

EiDA provides a computationally fast, analytically based, closed form method to extract dynamical connectivity information in a lossless manner from signals in high-dimensional time-series. Its application to fMRI data from a longitudinal rat ageing study demonstrated its advantages over conventional methods and allows a formal relationship between dynamical and informational metrics. This approach holds promise in enhancing the accuracy of potential neuromechanistic biomarkers for diseases or the assessment of intervention studies. Moreover, EiDA can be applied to task fMRI, for example in association with individual differences or to investigate the dynamic properties of the brain during a specific task. The two most straightforward application can be, for example, using continuous EiDA measures during the task and relating them to performance. Alternatively, it is be possible to apply Discrete EiDA to understand the different dynamic configuration of the brain during the task, and, again, relationship to performance. Furthermore, its potential extends beyond neuroscience, opening doors to various applications.

In summary, with this work we have introduced

- A full characterisation of the iPA matrix and its properties.
- An analytical and ultra-fast way of decomposing it.
- A new algorithm (Discrete EiDA) for the clustering of brain states, that can ease RAM requirements by orders of magnitude. Moreover, Discrete EiDA solves the “flipping sign” problem that was observed in LEiDA (see Section 2.8.1).
- A 2D embedding of dFC and two new measures for the continuous analysis of the connectivity patterns.

Utilising this methodology, we discovered multiple sensitive markers of the ageing brain:

- The average position, the first eigenvalue of the iPA matrix, which is related to the total quota of connectivity and the informational complexity of the iPA matrix.
- The dwell time and fractional occurrence of the brain states, which yield the highest significance and which inform us about the dynamic composition of the changes in connectivity patterns.
- A newly introduced measure, spectral metastability, which can be computed both in the overall recording and in the separate Discrete EiDA modes. This measure is more general than Kuramoto metastability.

These newly introduced measures are not only more sensitive than static measures but also provide deeper insights into the dynamics of the brain.

Finally, we emphasise that all these concepts may be extended to any square symmetric dFC matrix, after the choice of an adequate number of eigenvectors, which may not necessarily be two, making EiDA a general and comprehensive approach for dynamic functional connectivity.

## Supplementary Material

Supplementary Material Statistics

Supplementary Figures

## Data Availability

The code for EiDA, and the scripts to produce all the figures of this paper are available at www.github.com/alteriis/EiDA.
