# Supplementary material for: EiDA: A lossless approach for dynamic functional connectivity; application to fMRI data of a model of ageing
Source: Imaging Neurosci (Camb). 2024 Mar 22;2:imag-2-00113. doi: 10.1162/imag_a_00113 (PMC11801787; doi:10.1162/imag_a_00113)
Supplement: Supplementary Material Statistics [file imag_a_00113-supplementary_material.pdf]

measure: FC index for ipl matrix
N=30
Nonparametric ANOVA (kruskal wallis) p-value: 9.330767e-09

Source	SS	df	MS	Chi-sq	Prob>Chi-sq
{'Columns'}	{[4.8729e+04]}	{[3]}	{[1.6243e+04]}	{[40.2719]}	{[9.3308e-09]}
{'Error' }	{[9.5261e+04]}	{[116]}	{[821.2155]}	{0×0 double}	{0×0 double }
{'Total' }	{[143990]}	{[119]}	{0×0 double }	{0×0 double}	{0×0 double }

Post Hoc Wilcoxon Pairwise Comparisons with Benjamini-Hochberg Correction:
Month 3 vs 5, p=8.944301e-04, reject=1
Month 3 vs 11, p=4.285686e-06, reject=1
Month 3 vs 17, p=1.238080e-05, reject=1
Month 5 vs 11, p=7.271050e-03, reject=1
Month 5 vs 17, p=5.319684e-03, reject=1
Month 11 vs 17, p=4.048347e-01, reject=0

Means: Month 3: 1.743940e+01 5: 1.432518e+01 11: 1.238604e+01 17: 1.202920e+01
Standard Devs: 3: 3.783279e+00 5: 2.822229e+00 11: 2.334583e+00 17: 2.665591e+00

Gaussianity Anderson-Darling Tests (1=reject):
Month 3: 0 Month 5: 0 Month 11: 1 Month 17: 1

~~~~~~~~~~~~~~~~~~~~~~~~~~~~~~~~~~

measure: FC index correlation matrix
N=30
Nonparametric ANOVA (kruskal wallis) p-value: 9.854890e-09

Source	SS	df	MS	Chi-sq	Prob>Chi-sq
{'Columns'}	{[4.8594e+04]}	{[3]}	{[1.6198e+04]}	{[40.1599]}	{[9.8549e-09]}
{'Error' }	{[9.5396e+04]}	{[116]}	{[822.3833]}	{0×0 double}	{0×0 double }
{'Total' }	{[143990]}	{[119]}	{0×0 double }	{0×0 double}	{0×0 double }

Post Hoc Wilcoxon Pairwise Comparisons with Benjamini-Hochberg Correction:
Month 3 vs 5, p=1.035681e-03, reject=1
Month 3 vs 11, p=2.603328e-06, reject=1
Month 3 vs 17, p=8.466082e-06, reject=1
Month 5 vs 11, p=2.584559e-03, reject=1
Month 5 vs 17, p=1.592702e-03, reject=1
Month 11 vs 17, p=5.038330e-01, reject=0

Means: Month 3: 1.969628e+01 5: 1.678065e+01 11: 1.408462e+01 17: 1.367809e+01
Standard Devs: 3: 3.878135e+00 5: 3.350128e+00 11: 2.939263e+00 17: 3.123053e+00

Gaussianity Anderson-Darling Tests (1=reject):
Month 3: 0 Month 5: 0 Month 11: 1 Month 17: 1
correlation = 9.723250e-01,significance=1
~~~~~~~~~~~~~~~~~~~~~~~~~~~~~~~~~~

~~~~~~~~~~~~~~~~~~~~~~~~~~~~~~~~~~~
measure: speed 1st eigenvector
N=30
Nonparametric ANOVA (kruskal wallis) p-value: 1.366842e-05

Source	SS	df	MS	Chi-sq	Prob>Chi-sq
{'Columns'}	{[3.0556e+04]}	{[3]}	{[1.0185e+04]}	{[25.2532]}	{[1.3668e-05]}
{'Error' }	{[1.1343e+05]}	{[116]}	{[977.8764]}	{0×0 double}	{0×0 double }
{'Total' }	{[143990]}	{[119]}	{0×0 double }	{0×0 double}	{0×0 double }

Post Hoc Wilcoxon Pairwise Comparisons with Benjamini-Hochberg Correction:
Month 3 vs 5, p=3.682613e-02, reject=0
Month 3 vs 11, p=3.515237e-06, reject=1
Month 3 vs 17, p=1.359477e-04, reject=1
Month 5 vs 11, p=5.319684e-03, reject=1
Month 5 vs 17, p=1.254382e-01, reject=0
Month 11 vs 17, p=2.369362e-01, reject=0

Means: Month 3: 1.784098e-01 5: 1.903294e-01 11: 2.061423e-01 17: 1.996177e-01
Standard Devs: 3: 2.173233e-02 5: 2.096710e-02 11: 1.577545e-02 17: 2.208164e-02

Gaussianity Anderson-Darling Tests (1=reject):
Month 3: 1 Month 5: 0 Month 11: 0 Month 17: 0

~~~~~~~~~~~~~~~~~~~~~~~~~~~~~~~~~~~
measure: speed 2nd eigenvector
N=30
Nonparametric ANOVA (kruskal wallis) p-value: 2.089630e-01

Source	SS	df	MS	Chi-sq	Prob>Chi-sq
{'Columns'}	{[5.4905e+03]}	{[3]}	{[1.8302e+03]}	{[4.5376]}	{[0.2090]}
{'Error' }	{[1.3850e+05]}	{[116]}	{[1.1940e+03]}	{0×0 double}	{0×0 double}
{'Total' }	{[143990]}	{[119]}	{0×0 double }	{0×0 double}	{0×0 double}

Post Hoc Wilcoxon Pairwise Comparisons with Benjamini-Hochberg Correction:
Month 3 vs 5, p=1.915221e-01, reject=0
Month 3 vs 11, p=3.000989e-02, reject=0
Month 3 vs 17, p=1.174811e-02, reject=0
Month 5 vs 11, p=6.732798e-01, reject=0
Month 5 vs 17, p=5.304401e-01, reject=0
Month 11 vs 17, p=9.589902e-01, reject=0

Means: Month 3: 2.449856e-01 5: 2.519729e-01 11: 2.543254e-01 17: 2.533717e-01
Standard Devs: 3: 1.776172e-02 5: 1.282094e-02 11: 1.531508e-02 17: 1.304118e-02

Gaussianity Anderson-Darling Tests (1=reject):
Month 3: 0 Month 5: 0 Month 11: 0 Month 17: 0

~~~~~~~~~~~~~~~~~~~~~~~~~~~~~~~~~~~
measure: average first eigenvalue=spectral radius
N=30
Nonparametric ANOVA (kruskal wallis) p-value: 1.030758e-08

Source	SS	df	MS	Chi-sq	Prob>Chi-sq
{'Columns'}	{[4.8482e+04]}	{[3]}	{[1.6161e+04]}	{[40.0679]}	{[1.0308e-08]}
{'Error' }	{[9.5508e+04]}	{[116]}	{[823.3431]}	{0×0 double}	{0×0 double }
{'Total' }	{[143990]}	{[119]}	{0×0 double }	{0×0 double}	{0×0 double }

Post Hoc Wilcoxon Pairwise Comparisons with Benjamini-Hochberg Correction:
Month 3 vs 5, p=8.944301e-04, reject=1
Month 3 vs 11, p=4.729202e-06, reject=1
Month 3 vs 17, p=1.360111e-05, reject=1
Month 5 vs 11, p=3.854236e-03, reject=1
Month 5 vs 17, p=2.414704e-03, reject=1
Month 11 vs 17, p=5.440062e-01, reject=0
~~~~~~~~~~~~~~~~~~~~~~~~~~~~~~~~~~~

```
Means:  Month 3: 2.946121e+01   5: 2.805408e+01   11: 2.713789e+01   17: 2.702104e+01
Standard Devs:  3: 1.835784e+00   5: 1.276381e+00   11: 1.005053e+00   17: 1.076974e+00

Gaussianity Anderson-Darling Tests (1=reject):
Month 3: 0   Month 5: 0   Month 11: 0   Month 17: 0




~~~~~~~~~~~~~~~~~~~~~~~~~~~~~~~~~~~
measure: zip complexity
 N=30
Nonparametric ANOVA (kruskal wallis) p-value: 1.250180e-08

      Source             SS            df            MS              Chi-sq       Prob>Chi-sq
    ___________    ______________    _______    ______________    ____________    ______________

    {'Columns'}    {[4.8004e+04]}    {[  3]}    {[1.6001e+04]}    {[ 39.6725]}    {[1.2502e-08]}
    {'Error'  }    {[9.5986e+04]}    {[116]}    {[  827.4651]}    {0×0 double}    {0×0 double  }
    {'Total'  }    {[1.4399e+05]}    {[119]}    {0×0 double  }    {0×0 double}    {0×0 double  }


Post Hoc Wilcoxon Pairwise Comparisons with Benjamini-Hochberg Correction:
Month 3 vs 5, p=1.286631e-03, reject=1
Month 3 vs 11, p=1.360111e-05, reject=1
Month 3 vs 17, p=3.724265e-05, reject=1
Month 5 vs 11, p=3.854236e-03, reject=1
Month 5 vs 17, p=1.286631e-03, reject=1
Month 11 vs 17, p=3.184778e-01, reject=0

Means:  Month 3: 2.509426e+06   5: 2512707   11: 2.514419e+06   17: 2.514659e+06
Standard Devs:  3: 4.898115e+03   5: 2.640916e+03   11: 1.793370e+03   17: 1.891798e+03

Gaussianity Anderson-Darling Tests (1=reject):
Month 3: 0   Month 5: 1 Month 11: 1 Month 17: 1


~~~~~~~~~~~~~~~~~~~~~~~~~~~~~~~~~~~
measure: std reconf speed
 N=30
Nonparametric ANOVA (kruskal wallis) p-value: 4.638456e-08

      Source             SS            df            MS              Chi-sq       Prob>Chi-sq
    ___________    ______________    _______    ______________    ____________    ______________

    {'Columns'}    {[4.4750e+04]}    {[  3]}    {[1.4917e+04]}    {[ 36.9835]}    {[4.6385e-08]}
    {'Error'  }    {[9.9240e+04]}    {[116]}    {[  855.5167]}    {0×0 double}    {0×0 double  }
    {'Total'  }    {[    143990]}    {[119]}    {0×0 double  }    {0×0 double}    {0×0 double  }


Post Hoc Wilcoxon Pairwise Comparisons with Benjamini-Hochberg Correction:
Month 3 vs 5, p=6.156406e-04, reject=1
Month 3 vs 11, p=1.024633e-05, reject=1
Month 3 vs 17, p=1.639446e-05, reject=1
Month 5 vs 11, p=6.268281e-02, reject=0
Month 5 vs 17, p=2.105260e-03, reject=1
Month 11 vs 17, p=1.413895e-01, reject=0

Means:  Month 3: 1.333286e-01   5: 1.188397e-01   11: 1.115129e-01   17: 1.062699e-01
Standard Devs:  3: 1.524897e-02   5: 1.618577e-02   11: 1.536319e-02   17: 1.397473e-02

Gaussianity Anderson-Darling Tests (1=reject):
Month 3: 0   Month 5: 0   Month 11: 1   Month 17: 1



                              ~~~~~~~~~~~~~~~~~~~~~~~~~~~~~~~~~~~
measure: mean reconf speed
 N=30
Nonparametric ANOVA (kruskal wallis) p-value: 1.669891e-04

      Source             SS            df            MS              Chi-sq       Prob>Chi-sq
    ___________    ______________    _______    ______________    ____________    ______________

    {'Columns'}    {[2.4241e+04]}    {[  3]}    {[8.0805e+03]}    {[ 20.0343]}    {[1.6699e-04]}
    {'Error'  }    {[1.1975e+05]}    {[116]}    {[1.0323e+03]}    {0×0 double}    {0×0 double  }
    {'Total'  }    {[    143990]}    {[119]}    {0×0 double  }    {0×0 double}    {0×0 double  }
```

Post Hoc Wilcoxon Pairwise Comparisons with Benjamini-Hochberg Correction:
Month 3 vs 5, p=1.020107e-01, reject=0
Month 3 vs 11, p=2.596713e-05, reject=1
Month 3 vs 17, p=4.195510e-04, reject=1
Month 5 vs 11, p=1.397456e-02, reject=1
Month 5 vs 17, p=1.528607e-01, reject=0
Month 11 vs 17, p=5.170479e-01, reject=0

Means: Month 3: 2.212638e-01 5: 2.321094e-01 11: 2.424817e-01 17: 2.384687e-01
Standard Devs: 3: 2.139190e-02 5: 1.632248e-02 11: 1.028617e-02 17: 1.455955e-02

Gaussianity Anderson-Darling Tests (1=reject):
Month 3: 1 Month 5: 0 Month 11: 0 Month 17: 0

~~~~~~~~~~~~~~~~~~~~~~~~~~~~~~~~~~~
measure: metastability
N=30
Nonparametric ANOVA (kruskal wallis) p-value: 4.437007e-13

Source	SS	df	MS	Chi-sq	Prob>Chi-sq
{'Columns'}	{[7.3292e+04]}	{[3]}	{[2.4431e+04]}	{[60.5717]}	{[4.4370e-13]}
{'Error' }	{[7.0698e+04]}	{[116]}	{[609.4672]}	{0×0 double}	{0×0 double }
{'Total' }	{[143990]}	{[119]}	{0×0 double }	{0×0 double}	{0×0 double }

Post Hoc Wilcoxon Pairwise Comparisons with Benjamini-Hochberg Correction:
Month 3 vs 5, p=5.792446e-05, reject=1
Month 3 vs 11, p=1.734398e-06, reject=1
Month 3 vs 17, p=6.339136e-06, reject=1
Month 5 vs 11, p=2.830789e-04, reject=1
Month 5 vs 17, p=4.071512e-05, reject=1
Month 11 vs 17, p=3.161765e-03, reject=1

Means: Month 3: 1.303540e-02 5: 7.771787e-03 11: 4.630907e-03 17: 3.795565e-03
Standard Devs: 3: 6.643573e-03 5: 4.018835e-03 11: 2.155167e-03 17: 2.661123e-03

Gaussianity Anderson-Darling Tests (1=reject):
Month 3: 0 Month 5: 1 Month 11: 1 Month 17: 1

~~~~~~~~~~~~~~~~~~~~~~~~~~~~~~~~~~~
measure: spectral metastability
N=30
Nonparametric ANOVA (kruskal wallis) p-value: 7.790465e-07

Source	SS	df	MS	Chi-sq	Prob>Chi-sq
{'Columns'}	{[3.7728e+04]}	{[3]}	{[1.2576e+04]}	{[31.1799]}	{[7.7905e-07]}
{'Error' }	{[1.0626e+05]}	{[116]}	{[916.0546]}	{0×0 double}	{0×0 double }
{'Total' }	{[143990]}	{[119]}	{0×0 double }	{0×0 double}	{0×0 double }

Post Hoc Wilcoxon Pairwise Comparisons with Benjamini-Hochberg Correction:
Month 3 vs 5, p=4.949805e-02, reject=0
Month 3 vs 11, p=3.405257e-05, reject=1
Month 3 vs 17, p=4.449337e-05, reject=1
Month 5 vs 11, p=1.286631e-03, reject=1
Month 5 vs 17, p=2.765274e-03, reject=1
Month 11 vs 17, p=7.655193e-01, reject=0

Means: Month 3: 3.236936e+00 5: 3.005686e+00 11: 2.653202e+00 17: 2.614077e+00
Standard Devs: 3: 4.379134e-01 5: 4.394796e-01 11: 4.375050e-01 17: 4.519052e-01

Gaussianity Anderson-Darling Tests (1=reject):
Month 3: 0 Month 5: 0 Month 11: 0 Month 17: 0

~~~~~~~~~~~~~~~~~~~~~~~~~~~~~~~~~~~
measure: fractional occurrence of cluster 1
N=30
Nonparametric ANOVA (kruskal wallis) p-value: 6.760030e-10

Source	SS	df	MS	Chi-sq	Prob>Chi-sq
{'Columns'}	{[5.5215e+04]}	{[3]}	{[1.8405e+04]}	{[45.6413]}	{[6.7600e-10]}
{'Error' }	{[8.8745e+04]}	{[116]}	{[765.0473]}	{0×0 double}	{0×0 double }
{'Total' }	{[143960]}	{[119]}	{0×0 double }	{0×0 double}	{0×0 double }

Post Hoc Wilcoxon Pairwise Comparisons with Benjamini-Hochberg Correction:
Month 3 vs 5, p=3.184394e-01, reject=0
Month 3 vs 11, p=6.361173e-01, reject=0
Month 3 vs 17, p=2.598580e-06, reject=1
Month 5 vs 11, p=7.342847e-01, reject=0
Month 5 vs 17, p=8.876318e-06, reject=1
Month 11 vs 17, p=1.637758e-05, reject=1

Means: Month 3: 4.552682e-01 5: 4.352490e-01 11: 4.273946e-01 17: 2.345785e-01
Standard Devs: 3: 1.079040e-01 5: 9.034125e-02 11: 8.729963e-02 17: 1.137359e-01

Gaussianity Anderson-Darling Tests (1=reject):
Month 3: 0 Month 5: 0 Month 11: 0 Month 17: 0

~~~~~~~~~~~~~~~~~~~~~~~~~~~~~~~~~~~
measure: avg duration of cluster 1
N=30
Nonparametric ANOVA (kruskal wallis) p-value: 1.487152e-10

Source	SS	df	MS	Chi-sq	Prob>Chi-sq
{'Columns'}	{[5.8966e+04]}	{[3]}	{[1.9655e+04]}	{[48.7326]}	{[1.4872e-10]}
{'Error' }	{[8.5023e+04]}	{[116]}	{[732.9542]}	{0×0 double}	{0×0 double }
{'Total' }	{[1.4399e+05]}	{[119]}	{0×0 double }	{0×0 double}	{0×0 double }

Post Hoc Wilcoxon Pairwise Comparisons with Benjamini-Hochberg Correction:
Month 3 vs 5, p=1.956922e-02, reject=1
Month 3 vs 11, p=1.708773e-03, reject=1
Month 3 vs 17, p=1.734398e-06, reject=1
Month 5 vs 11, p=7.521331e-02, reject=0
Month 5 vs 17, p=7.513662e-05, reject=1
Month 11 vs 17, p=1.477276e-04, reject=1

Means: Month 3: 4.339735e+01 5: 2.676665e+01 11: 2.234757e+01 17: 1.240051e+01
Standard Devs: 3: 2.824505e+01 5: 1.112871e+01 11: 5.111319e+00 17: 8.387121e+00

Gaussianity Anderson-Darling Tests (1=reject):
Month 3: 1 Month 5: 1 Month 11: 0 Month 17: 0

~~~~~~~~~~~~~~~~~~~~~~~~~~~~~~~~~~~
measure: metastability of cluster 1
N=30
Nonparametric ANOVA (kruskal wallis) p-value: 2.185427e-11

Source	SS	df	MS	Chi-sq	Prob>Chi-sq
{'Columns'}	{[63697]}	{[3]}	{[2.1232e+04]}	{[52.6421]}	{[2.1854e-11]}
{'Error' }	{[80293]}	{[116]}	{[692.1810]}	{0×0 double}	{0×0 double }
{'Total' }	{[143990]}	{[119]}	{0×0 double }	{0×0 double}	{0×0 double }

Post Hoc Wilcoxon Pairwise Comparisons with Benjamini-Hochberg Correction:
Month 3 vs 5, p=4.652826e-01, reject=0
Month 3 vs 11, p=3.064999e-04, reject=1
Month 3 vs 17, p=1.920921e-06, reject=1
Month 5 vs 11, p=2.765274e-03, reject=1
Month 5 vs 17, p=8.466082e-06, reject=1
Month 11 vs 17, p=5.306992e-05, reject=1
~~~~~~~~~~~~~~~~~~~~~~~~~~~~~~~~~~~

Means: Month 3: 3.182195e+00   5: 3.063381e+00   11: 2.734559e+00   17: 2.189846e+00
Standard Devs: 3: 4.141962e-01   5: 4.858451e-01   11: 4.569723e-01   17: 3.509641e-01

Gaussianity Anderson-Darling Tests (1=reject):
Month 3: 0   Month 5: 0   Month 11: 1   Month 17: 1

~~~~~~~~~~~~~~~~~~~~~~~~~~~~~~~~~~~
measure: fractional occurrence of cluster 2
N=30
Nonparametric ANOVA (kruskal wallis) p-value: 1.173425e-12

Source	SS	df	MS	Chi-sq	Prob>Chi-sq
{'Columns'}	{[7.0884e+04]}	{[3]}	{[2.3628e+04]}	{[58.5945]}	{[1.1734e-12]}
{'Error' }	{[7.3075e+04]}	{[116]}	{[629.9579]}	{0×0 double}	{0×0 double }
{'Total' }	{[1.4396e+05]}	{[119]}	{0×0 double }	{0×0 double}	{0×0 double }

Post Hoc Wilcoxon Pairwise Comparisons with Benjamini-Hochberg Correction:
Month 3 vs 5, p=8.218217e-02, reject=0
Month 3 vs 11, p=2.871658e-06, reject=1
Month 3 vs 17, p=2.176651e-03, reject=1
Month 5 vs 11, p=4.278194e-06, reject=1
Month 5 vs 17, p=1.131716e-01, reject=0
Month 11 vs 17, p=4.280690e-06, reject=1

Means: Month 3: 4.518199e-01   5: 4.117816e-01   11: 1.847701e-01   17: 3.698276e-01
Standard Devs: 3: 1.056049e-01   5: 1.018370e-01   11: 1.016747e-01   17: 6.536700e-02

Gaussianity Anderson-Darling Tests (1=reject):
Month 3: 0. Month 5: 0   Month 11: 1   Month 17: 1

~~~~~~~~~~~~~~~~~~~~~~~~~~~~~~~~~~~
measure: avg duration of cluster 2
N=30
Nonparametric ANOVA (kruskal wallis) p-value: 1.477730e-15

Source	SS	df	MS	Chi-sq	Prob>Chi-sq
{'Columns'}	{[8.7301e+04]}	{[3]}	{[2.9100e+04]}	{[72.1509]}	{[1.4777e-15]}
{'Error' }	{[5.6686e+04]}	{[116]}	{[488.6763]}	{0×0 double}	{0×0 double }
{'Total' }	{[1.4399e+05]}	{[119]}	{0×0 double }	{0×0 double}	{0×0 double }

Post Hoc Wilcoxon Pairwise Comparisons with Benjamini-Hochberg Correction:
Month 3 vs 5, p=3.378854e-03, reject=1
Month 3 vs 11, p=2.126636e-06, reject=1
Month 3 vs 17, p=7.690859e-06, reject=1
Month 5 vs 11, p=1.920921e-06, reject=1
Month 5 vs 17, p=6.156406e-04, reject=1
Month 11 vs 17, p=2.370448e-05, reject=1

Means: Month 3: 4.339217e+01   5: 2.658974e+01   11: 9.669624e+00   17: 1.791753e+01
Standard Devs: 3: 2.655078e+01   5: 1.339043e+01   11: 4.336543e+00   17: 5.271858e+00

Gaussianity Anderson-Darling Tests (1=reject):
Month 3: 1   Month 5:1   Month 11: 1   Month 17: 1

~~~~~~~~~~~~~~~~~~~~~~~~~~~~~~~~~~~
measure: metastability of cluster 2
N=30
Nonparametric ANOVA (kruskal wallis) p-value: 4.421238e-07

Source	SS	df	MS	Chi-sq	Prob>Chi-sq
{'Columns'}	{[3.9140e+04]}	{[3]}	{[1.3047e+04]}	{[32.3475]}	{[4.4212e-07]}
{'Error' }	{[1.0485e+05]}	{[116]}	{[903.8753]}	{0×0 double}	{0×0 double }
{'Total' }	{[143990]}	{[119]}	{0×0 double }	{0×0 double}	{0×0 double }
~~~~~~~~~~~~~~~~~~~~~~~~~~~~~~~~~~~

Post Hoc Wilcoxon Pairwise Comparisons with Benjamini-Hochberg Correction:
Month 3 vs 5, p=2.702916e-02, reject=1
Month 3 vs 11, p=2.163022e-05, reject=1
Month 3 vs 17, p=4.449337e-05, reject=1
Month 5 vs 11, p=1.149922e-04, reject=1
Month 5 vs 17, p=3.161765e-03, reject=1
Month 11 vs 17, p=5.038330e-01, reject=0

Means: Month 3: 2.957670e+00   5: 2.753671e+00   11: 2.376982e+00   17: 2.295182e+00
Standard Devs: 3: 3.843105e-01   5: 4.517109e-01   11: 4.039355e-01   17: 5.554944e-01

Gaussianity Anderson-Darling Tests (1=reject):
Month 3: 0   Month 5: 0   Month 11: 0   Month 17: 0

~~~~~~~~~~~~~~~~~~~~~~~~~~~~~~~~~~~
measure: fractional occurrence of cluster 3
N=30
Nonparametric ANOVA (kruskal wallis) p-value: 1.236732e-16

Source	SS	df	MS	Chi-sq	Prob>Chi-sq
{'Columns'}	{[9.3374e+04]}	{[3]}	{[3.1125e+04]}	{[77.1777]}	{[1.2367e-16]}
{'Error' }	{[5.0599e+04]}	{[116]}	{[436.1980]}	{0×0 double}	{0×0 double }
{'Total' }	{[143973]}	{[119]}	{0×0 double }	{0×0 double}	{0×0 double }

Post Hoc Wilcoxon Pairwise Comparisons with Benjamini-Hochberg Correction:
Month 3 vs 5, p=6.414400e-03, reject=1
Month 3 vs 11, p=2.353421e-06, reject=1
Month 3 vs 17, p=1.732216e-06, reject=1
Month 5 vs 11, p=4.071993e-06, reject=1
Month 5 vs 17, p=5.741856e-06, reject=1
Month 11 vs 17, p=4.112034e-01, reject=0

Means: Month 3: 9.291188e-02   5: 1.529693e-01   11: 3.878352e-01   17: 3.955939e-01
Standard Devs: 3: 9.955840e-02   5: 9.829296e-02   11: 1.224521e-01   17: 9.764673e-02

Gaussianity Anderson-Darling Tests (1=reject):
Warning: P is less than the smallest tabulated value, returning 0.0005.
> In adtest (line 281)
In nonpar_anova_wilcoxon (line 65)
In figure_5_NEW_ALGO (line 96)
Month 3: 1   Month 5: 0   Month 11: 0   Month 17: 0

~~~~~~~~~~~~~~~~~~~~~~~~~~~~~~~~~~~
measure: avg duration of cluster 3
N=30
Nonparametric ANOVA (kruskal wallis) p-value: 2.323208e-15

Source	SS	df	MS	Chi-sq	Prob>Chi-sq
{'Columns'}	{[8.6181e+04]}	{[3]}	{[2.8727e+04]}	{[71.2335]}	{[2.3232e-15]}
{'Error' }	{[5.7789e+04]}	{[116]}	{[498.1843]}	{0×0 double}	{0×0 double }
{'Total' }	{[143970]}	{[119]}	{0×0 double }	{0×0 double}	{0×0 double }

Post Hoc Wilcoxon Pairwise Comparisons with Benjamini-Hochberg Correction:
Month 3 vs 5, p=4.114031e-03, reject=1
Month 3 vs 11, p=3.515237e-06, reject=1
Month 3 vs 17, p=1.734398e-06, reject=1
Month 5 vs 11, p=3.724265e-05, reject=1
Month 5 vs 17, p=1.972948e-05, reject=1
Month 11 vs 17, p=5.716458e-01, reject=0

Means: Month 3: 6.379724e+00   5: 9.145384e+00   11: 1.904508e+01   17: 1.925484e+01
Standard Devs: 3: 4.153914e+00   5: 4.676275e+00   11: 7.363499e+00   17: 8.649783e+00

Gaussianity Anderson-Darling Tests (1=reject):
Month 3: 1   Month 5: 1   Month 11: 1   Month 17: 1

~~~~~~~~~~~~~~~~~~~~~~~~~~~~~~~~~~~
measure: metastability of cluster 3
N=29
Nonparametric ANOVA (kruskal wallis) p-value: 5.250033e-04

Source	SS	df	MS	Chi-sq	Prob>Chi-sq
{'Columns'}	{[1.9936e+04]}	{[3]}	{[6.6454e+03]}	{[17.6272]}	{[5.2500e-04]}
{'Error' }	{[1.1013e+05]}	{[112]}	{[983.2919]}	{0×0 double}	{0×0 double }
{'Total' }	{[130065]}	{[115]}	{0×0 double }	{0×0 double}	{0×0 double }

Post Hoc Wilcoxon Pairwise Comparisons with Benjamini-Hochberg Correction:
Month 3 vs 5, p=1.697296e-01, reject=0
Month 3 vs 11, p=1.765513e-01, reject=0
Month 3 vs 17, p=6.231683e-03, reject=1
Month 5 vs 11, p=2.128611e-02, reject=1
Month 5 vs 17, p=1.908042e-01, reject=0
Month 11 vs 17, p=2.085903e-04, reject=1

Means: Month 3: 2.329107e+00 5: 2.482088e+00 11: 2.202171e+00 17: 2.648467e+00
Standard Devs: 3: 5.089839e-01 5: 4.951227e-01 11: 3.080878e-01 17: 3.898462e-01

Gaussianity Anderson-Darling Tests (1=reject):
Month 3: 0 Month 5: 0 Month 11: 0 Month 17: 0

~~~~~~~~~~~~~~~~~~~~~~~~~~~~~~~~~~~

measure: mean of FCD matrix
N=30
Nonparametric ANOVA (kruskal wallis) p-value: 6.079821e-09

Source	SS	df	MS	Chi-sq	Prob>Chi-sq
{'Columns'}	{[4.9790e+04]}	{[3]}	{[1.6597e+04]}	{[41.1491]}	{[6.0798e-09]}
{'Error' }	{[9.4200e+04]}	{[116]}	{[812.0649]}	{0×0 double}	{0×0 double }
{'Total' }	{[143990]}	{[119]}	{0×0 double }	{0×0 double}	{0×0 double }

Post Hoc Wilcoxon Pairwise Comparisons with Benjamini-Hochberg Correction:
Month 3 vs 5, p=6.639213e-04, reject=1
Month 3 vs 11, p=2.353421e-06, reject=1
Month 3 vs 17, p=1.972948e-05, reject=1
Month 5 vs 11, p=2.255124e-03, reject=1
Month 5 vs 17, p=1.044440e-02, reject=1
Month 11 vs 17, p=7.035637e-01, reject=0

Means: Month 3: 8.046764e-01 5: 8.674876e-01 11: 9.049782e-01 17: 9.045038e-01
Standard Devs: 3: 7.769199e-02 5: 5.165891e-02 11: 4.159583e-02 17: 5.350391e-02

Gaussianity Anderson-Darling Tests (1=reject):
Month 3: 0 Month 5: 1 Month 11: 1 Month 17: 1

~~~~~~~~~~~~~~~~~~~~~~~~~~~~~~~~~~~
measure: FCD variance
N=30
Nonparametric ANOVA (kruskal wallis) p-value: 3.597948e-09

Source	SS	df	MS	Chi-sq	Prob>Chi-sq
{'Columns'}	{[5.1090e+04]}	{[3]}	{[1.7030e+04]}	{[42.2230]}	{[3.5979e-09]}
{'Error' }	{[9.2900e+04]}	{[116]}	{[800.8638]}	{0×0 double}	{0×0 double }
{'Total' }	{[143990]}	{[119]}	{0×0 double }	{0×0 double}	{0×0 double }

Post Hoc Wilcoxon Pairwise Comparisons with Benjamini-Hochberg Correction:
Month 3 vs 5, p=1.832580e-03, reject=1
Month 3 vs 11, p=2.603328e-06, reject=1
Month 3 vs 17, p=3.405257e-05, reject=1
Month 5 vs 11, p=6.639213e-04, reject=1
~~~~~~~~~~~~~~~~~~~~~~~~~~~~~~~~~~~

```
Month 5 vs 17, p=2.957462e-03, reject=1
Month 11 vs 17, p=5.577427e-01, reject=0

Means:  Month 3: 1.517248e-01   5: 1.323355e-01   11: 1.125898e-01   17: 1.129170e-01
Standard Devs:  3: 2.564218e-02   5: 2.296172e-02   11: 1.844742e-02   17: 2.466295e-02

Gaussianity Anderson-Darling Tests (1=reject):
Month 3: 0   Month 5: 0   Month 11: 1   Month 17: 1
```
