# Supplementary material for: EiDA: A lossless approach for dynamic functional connectivity; application to fMRI data of a model of ageing
Source: Imaging Neurosci (Camb). 2024 Mar 22;2:imag-2-00113. doi: 10.1162/imag_a_00113 (PMC11801787; doi:10.1162/imag_a_00113)
Supplement: Supplementary Figures [file imag_a_00113-supplementary_figures.pdf]

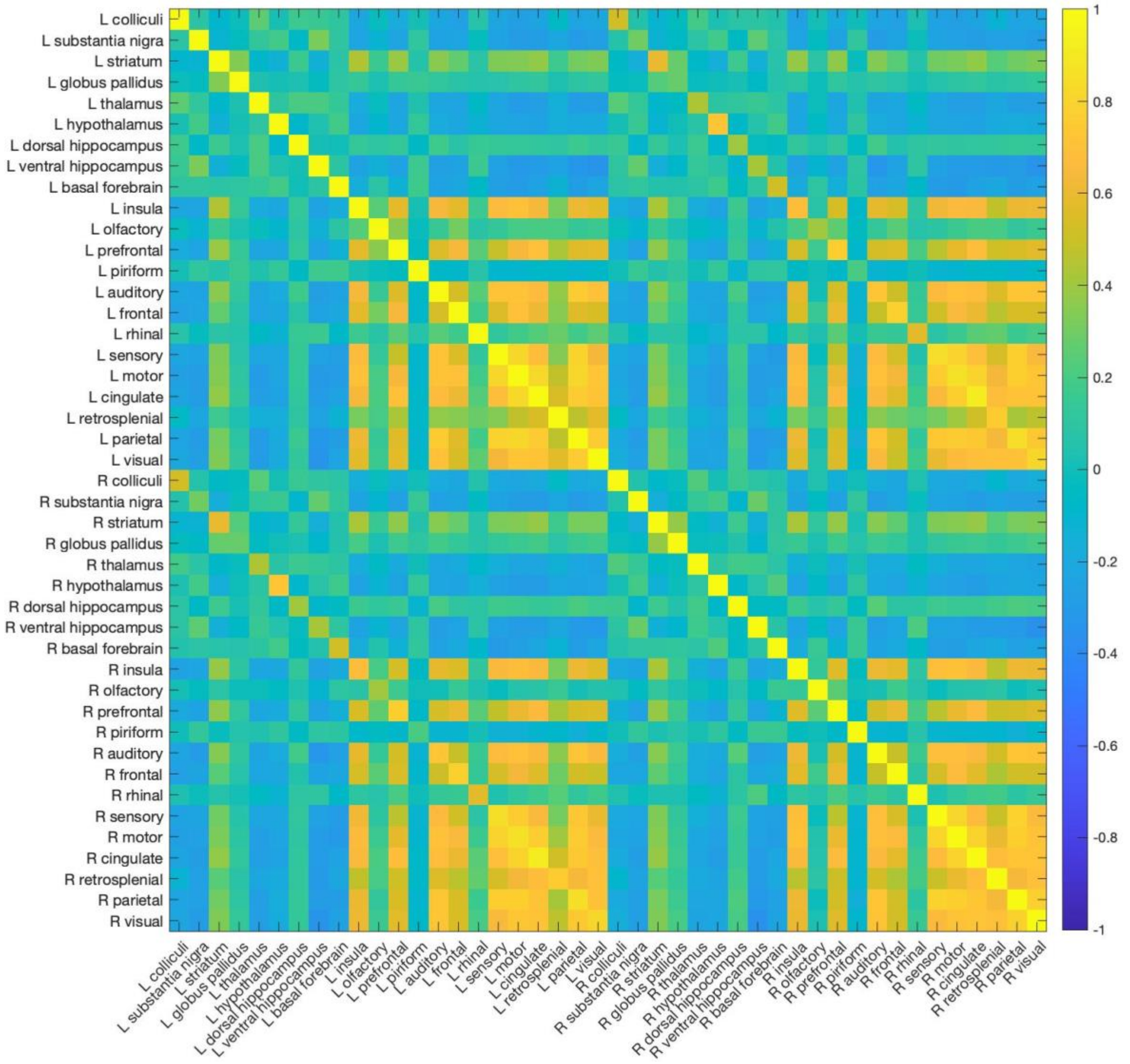

Figure S1: the first centroid outputted by the Discrete EiDA algorithm, in the age group of 3 months

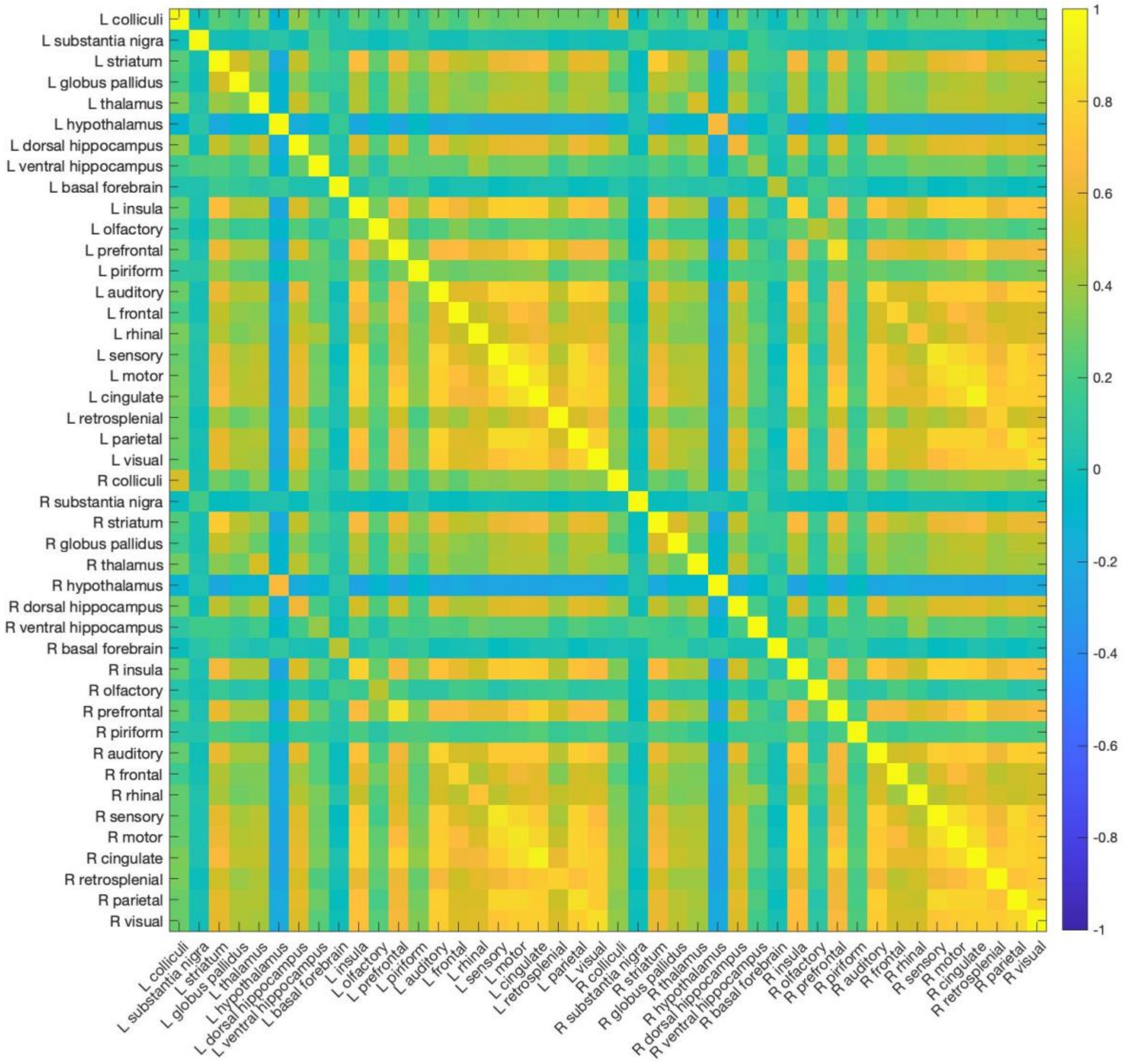

Figure S2: the second centroid outputted by the Discrete EiDA algorithm, in the age group of 3 months

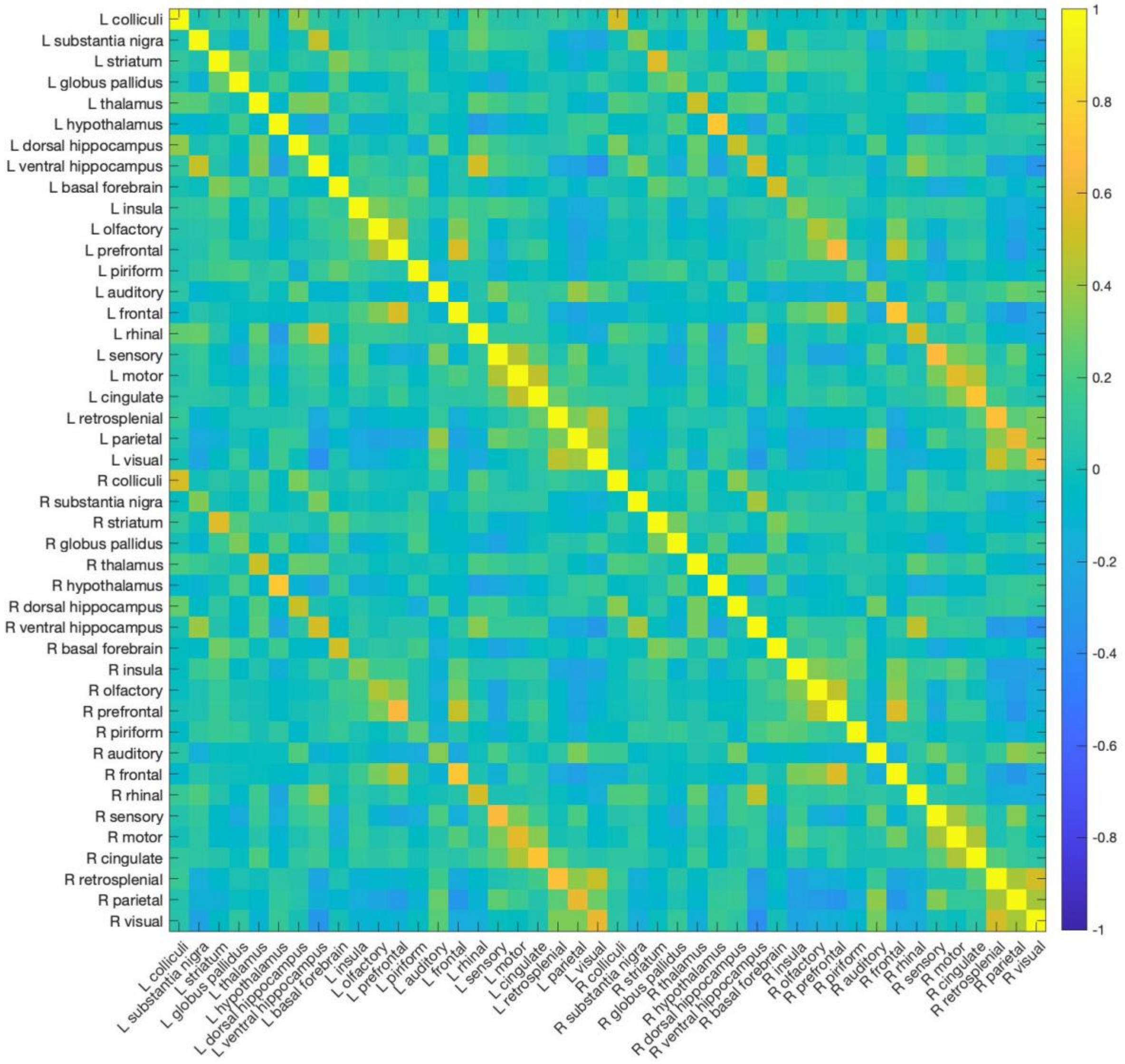

Figure S3: the third centroid outputted by the Discrete EiDA algorithm, in the age group of 3 months

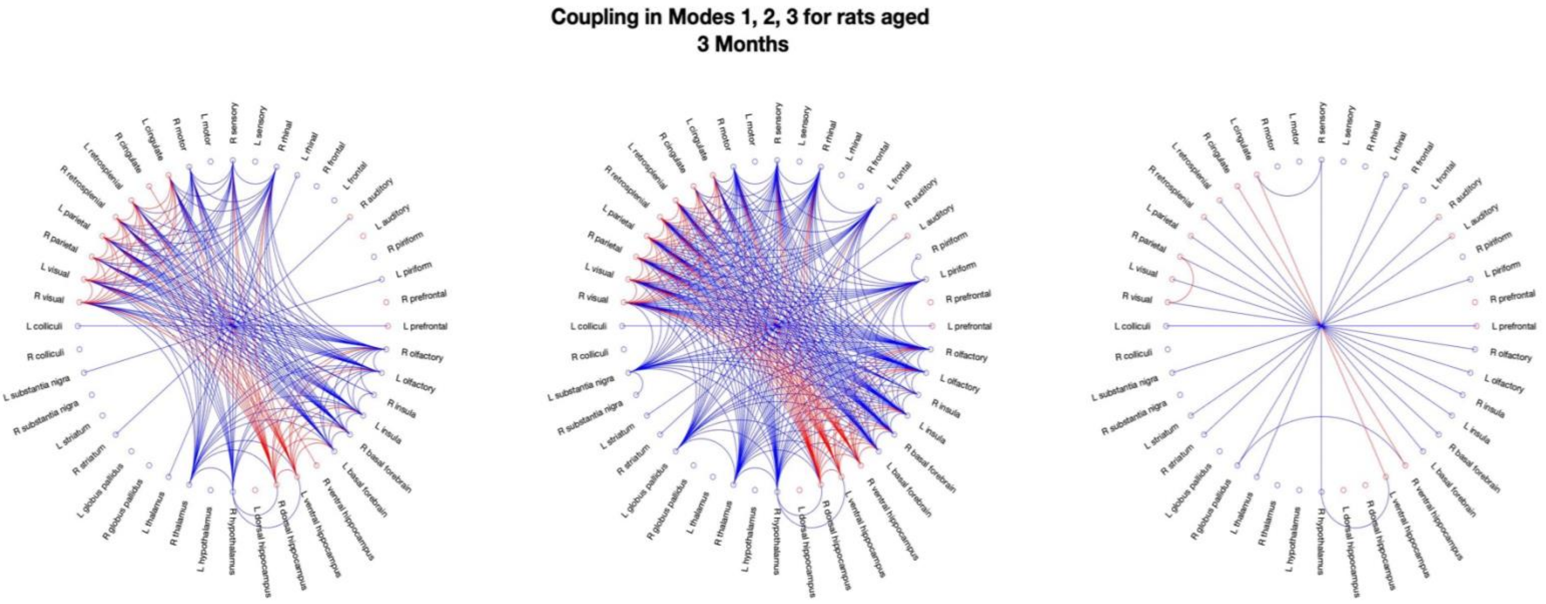

Figure S4: the three centroids at the age of 3 months, plotted as chord plots where only the connections higher than a threshold of 0.5 are plotted. Red connections belong to the Default Mode Network

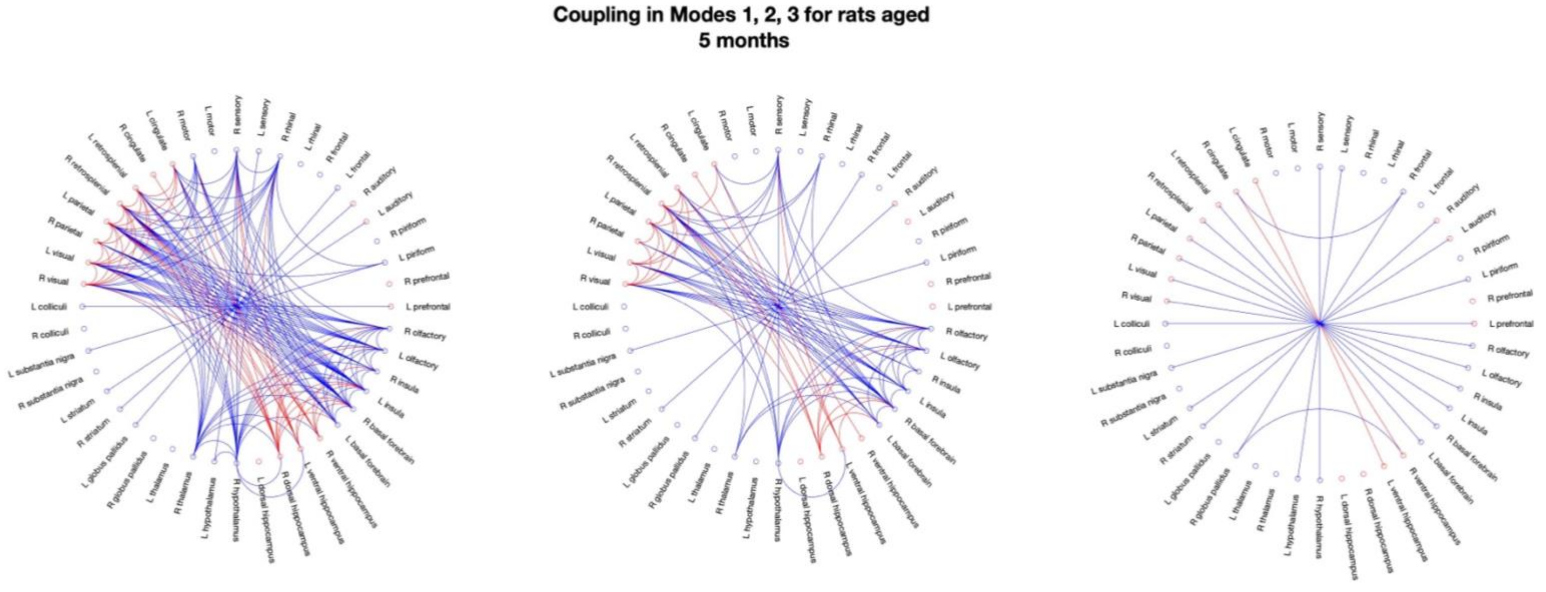

Figure S5: the three centroids at the age of 5 months, plotted as chord plots where only the connections higher than a threshold of 0.5 are plotted. Red connections belong to the Default Mode Network

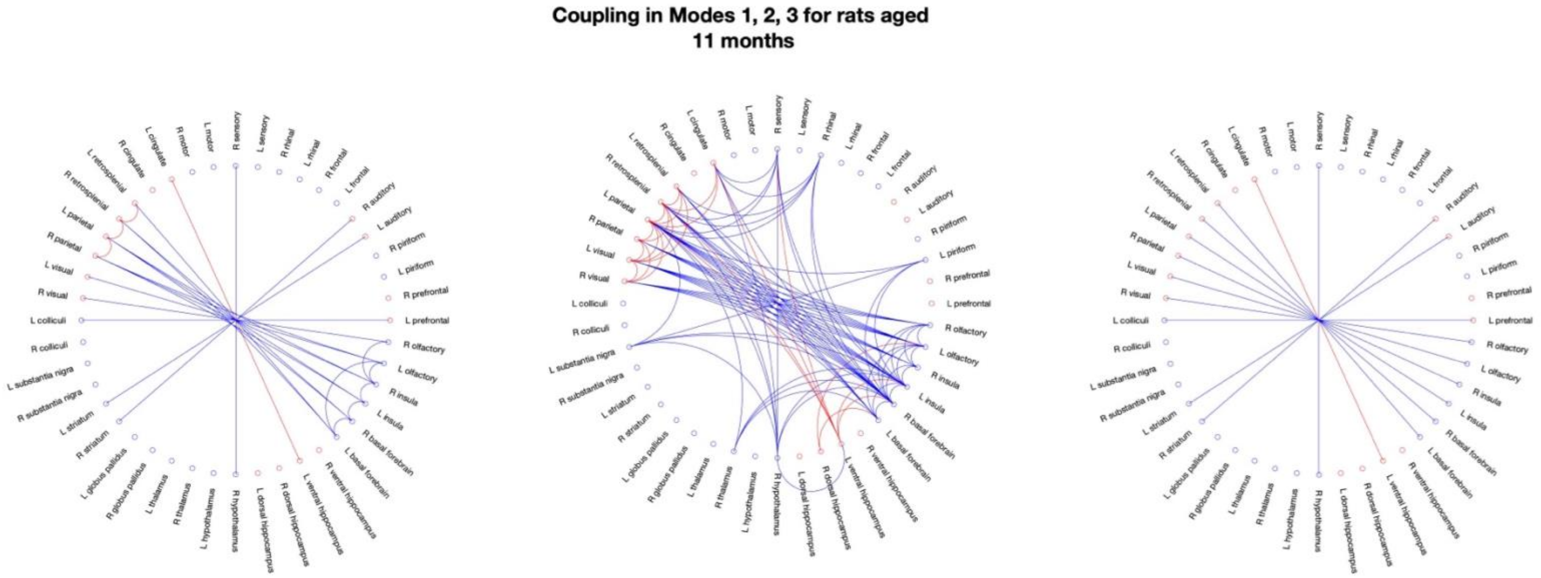

Figure S6: the three centroids at the age of 11 months, plotted as chord plots where only the connections higher than a threshold of 0.5 are plotted. Red connections belong to the Default Mode Network

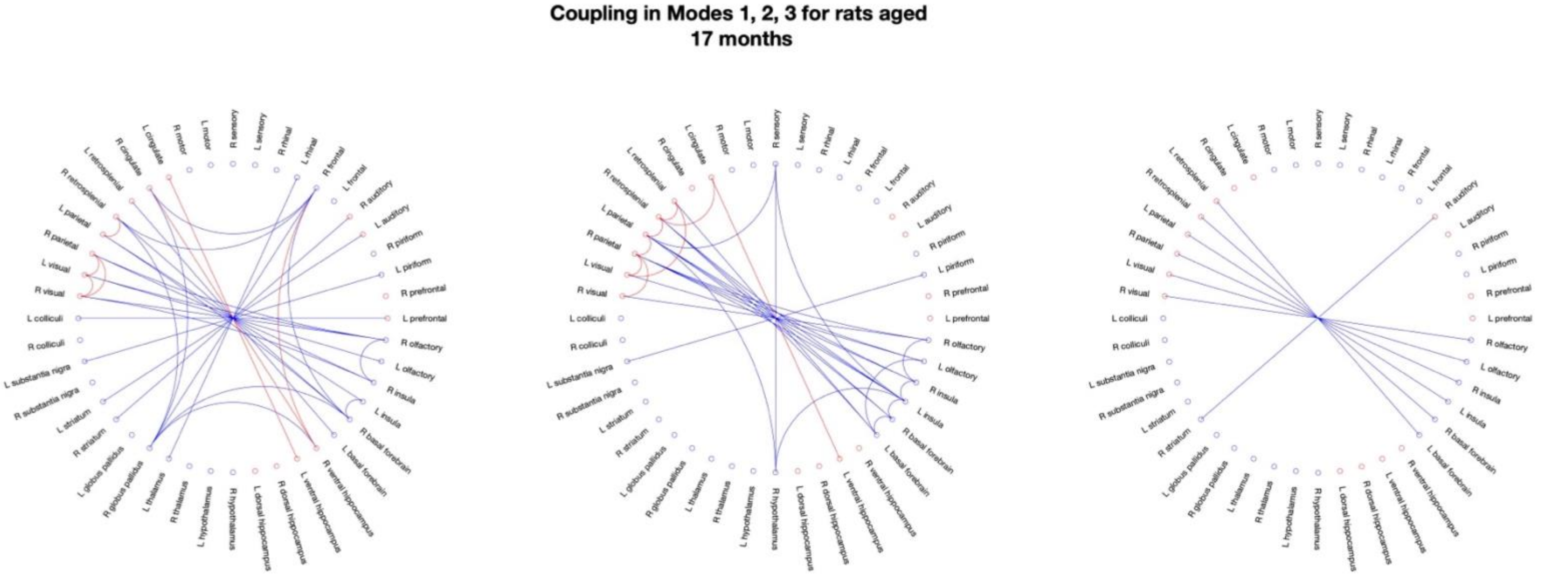

Figure S7: the three centroids at the age of 17 months, plotted as chord plots where only the connections higher than a threshold of 0.5 are plotted. Red connections belong to the Default Mode Network

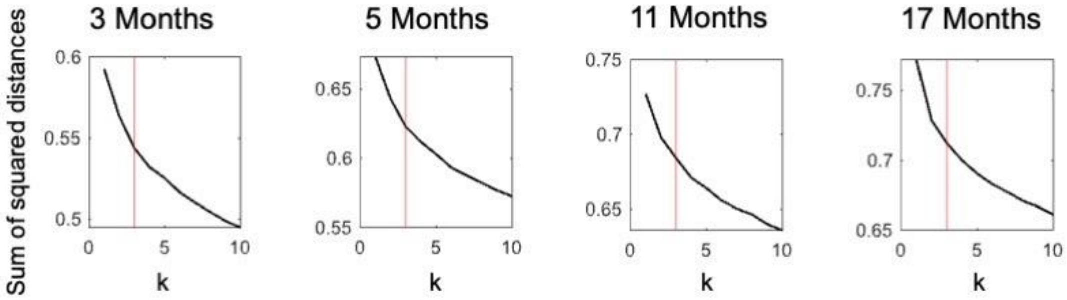

Figure S8: Elbow plots for the four age groups. X axis: number of clusters k. Y axis: sum of squared distances from the centroids. The red line indicates k=3, which is the designated number of clusters for the analysis.
